# Orthodontic treatment demand for fixed treatment and aligners among young adults in middle Europe and South America – a questionnaire study

**DOI:** 10.1186/s12903-024-04023-0

**Published:** 2024-03-02

**Authors:** Maciej Jedliński, Joyce Belfus, Marta Milona, Marta Mazur, Katarzyna Grocholewicz, Joanna Janiszewska-Olszowska

**Affiliations:** 1 Department of Interdisciplinary Dentistry, Pomeranian Medical University in Szczecin, al. Powstancow Wlkp. 72, 70-111 Szczecin, Poland; 2 Department of Oral and Maxillo-Facial Sciences, Sapienza University of Rome, Via Caserta 6, 00161 Rome, Italy; 3 Private Dental Practice, Adent- Ortodoncja i Stomatologia, ul. 4-go Marca 23G, 75-710 Koszalin, Poland; 4 Faculty of Dentistry, Universidad de los Andes, 7620001 Santiago, Chile; 5 Department of Hygiene and Epidemiology, Pomeranian Medical University, al. Powstancow Wlkp. 72, 70-111 Szczecin, Poland

**Keywords:** Orthodontic treatment need, Orthodontic treatment demand, Aligners, Fixed appliance, Patient’s experience, Questionnaire, Direct-to-consumer, Patient’s compliance, Europe, Latin America

## Abstract

**Background:**

Patients experiencing any malocclusion, may desire for treatment. However, there is no scientific information orthodontic treatment demand and the knowledge of young adults about orthodontic treatment. The aim of the study was to assess orthodontic treatment demand in young adults from Poland and Chile, their previous orthodontic experience and their knowledge on fixed and aligner orthodontic treatment.

**Methods:**

The target group comprised people aged 18–30. The sample size was estimated as above 400 for each country. The survey was carried out in Polish and Spanish within 3 months and consisted of 25 questions delivered via social media. Comparisons were made between countries, age subgroups and gender.

**Results:**

The response rate was 1,99%, what stands for 1092 responses, 670 from Chile and 422 from Poland, respectively. The percentage of young adults who were already treated was 42,9% in Poland and 25,0% in Chile. The ones planning to have orthodontic treatment within a year counted for 11,8% in Poland and 5,3% in Chile. Most young adults who want to be treated (20,6%) rely on doctor’s recommendation on type of appliance while 14,7% of all respondents are interested solely in aligners. Most respondents have heard about aligners (58%). Direct provider-to-customer service without a doctor is not acceptable, neither in Poland (85,1%) nor in Chile (64,8%). Most young adults provided incorrect answers referring various aspects of aligner treatment.

**Conclusions:**

In both countries, patients demand to be treated and monitored by the orthodontist. A high percentage of patients want to be treated exclusively with aligners. Direct-to-consumer orthodontics does not seem attractive to patients. Young adults do not have adequate knowledge referring to aligner treatment. Many people want to be treated despite a previous orthodontic treatment.

**Supplementary Information:**

The online version contains supplementary material available at 10.1186/s12903-024-04023-0.

## Background

Malocclusion has been recognized as a treatable chronic disability [1]. In many patients, mild occlusal discrepancies may be considered within the range of normal biologic variation without a need for treatment. In contrary, more severe malocclusions may have negative influence on orofacial function [2]. However, patient experiencing any malocclusion, may desire for treatment. Thus, it is important to distinguish between orthodontic treatment need and orthodontic treatment demand. Orthodontic treatment need is defined as urge for orthodontic intervention assessed professionally by a specialist; failure to provide orthodontic treatment could impair function of the masticatory system [3]. Orthodontic treatment demand is a subjective self-perceived orthodontic treatment desire – usually for esthetic or social reasons [4]. It is important to note that the demand for orthodontic treatment is not always consistent with the need for treatment, as factors such as cost, availability, and cultural attitudes can influence an individual’s decision to seek care. In the literature it is reported as ranging from 8,4 to 49% [5–7]. Orthodontic treatment demand depends on different factors, including gender, age or socioeconomic status [8]. In order to assess objective treatment need for the purpose of healthcare systems, in different countries numerous indexes have been developed such as index of orthodontic treatment need (IOTN), index of complexity, outcome, and need (ICON), dental aesthetic index (DAI) or treatment priority index (TPI). IOTN has gained the most popularity as it assesses both health treatment need (dental health component - DHC) and the esthetic component (AC), the aggregate of which indicates the need for orthodontic treatment [9]. Importantly, IOTN is the only indicator that has been found to be both repeatable and statistically quantifiable [9]. Moreover, it has been evidenced, that nowadays patients with esthetic motivations suffer higher psychosocial impacts, than those that should be treated solely for medical reasons [10]. It should be underlined, that orthodontic treatment need is characterized by lesser fluctuation than orthodontic treatment demand [10]. In the times of widespread availability of orthodontic services, it seems important to understand the characteristics of orthodontic treatment demand and the affecting factors.

According to a recent study published in British Dental Journal, young adults in their twenties and thirties tend to be especially interested in orthodontic treatment, as they often desire, but did not receive, orthodontic treatment during adolescence [11]. Now, these people can make fully independent decisions. On the other side, young people in different countries are subjected to direct marketing by orthodontic aligner companies, what may also affect their interest in treatment.

Beauty standards in Europe and South America have been shaped by different societal and historical influences. In Europe, beauty standards have long been set within society, with the ideal beauty standard heavily influenced by the ideal European figure, such as light skin, slim figure, gentle smile, and light-colored eyes [12]. However, in Latin America, beauty standards are complex and intersect with social background. Beautification and aesthetic medicine treatments are very popular there, which is visible in finical performance of the beauty industry, where Latin Americans stand out among emerging markets as spenders on beauty [13]. While beauty standards in both regions have some similarities, they are also shaped by unique cultural, social and historical contexts. It’s important to note that these standards are constantly evolving and being changed.

Digitalization is a global process [14] thus, it was considered worthwhile to study countries on opposite sides of the globe to see if the cultural phenomena of change associated with technological advances had similar exposures around the world. The decision to perform the study in Poland and in Chile was based both on the fact of globalization of products and service associated to orthodontic treatment and on the large geographical distance between countries on two distant continents. Moreover, it is important to underline that Poland and Chile are characterized by similar values of socio-economic and developmental indices. It is necessary to understand the background of the demand for orthodontic treatment in order to properly understand and obtain optimal compliance from patients who are motivated by different values than previous generations, and so to direct private practice activities to optimally meet the demand.

The authors did not find studies comparing orthodontic treatment demand of young adults from different continents. There is no scientific information on the knowledge and beliefs of young adults (as potential future orthodontic patients) about orthodontic treatment. It is not known, neither what type of treatment they desire nor what are the key factors that influence their decisions.

The aim of the study was to assess:

i) The orthodontic treatment demand in young adult population (between 18 to 30 years of age) in Poland and Chile.
ii) Previous orthodontic experience of the young adults.
iii) The knowledge and attitude of the young adults on fixed and aligner orthodontic treatment.
iv) The differences between Poland and Chile referring to the knowledge on orthodontic treatment.

## Material and methods

### Sample size adjustment

The sample size was estimated for both populations as above 400 people for each country, at the level of significance α = 0,05 [15].

### Ethical approval of the survey, informed consent to participate, and administration of the survey

This questionnaire study has been exempted from approval by bioethical committee of Pomeranian Medical University with decision reference number RWP/6546/2022P [Supplementary material S1]. Informed consent has been obtained from every participant before completion of a questionnaire. The questionnaire was preceded by the following information on the webpage: “Orthodontic treatment has become a part of everyday life for most of us. The survey is addressed to people who are not currently undergoing orthodontic treatment. Nowadays, orthodontics offers different types of appliances, i.e. fixed braces, removable acrylic braces or aligners (plastic transparent splints placed on the teeth). The purpose of the questionnaire is to provide information about your knowledge of current trends in orthodontic treatment, regardless of what you have already encountered with aligners and how anxious you are about aligner treatment in the future. Participation in the survey is addressed to adults and is voluntary. The survey is anonymous, Your email address will not be stored. If you agree to participate in the survey, please press the button below. Thank you for your cooperation.” After reading this information, the respondent had to turn to the next webpage, thereby expressing their willingness to participate. The target population were young adults between 18 and 30 years of age. The survey was carried out in Polish and Spanish languages within 3 months, from July to September 2022. First, the Polish version of questionnaire designed was resolved and assessed by a group of five experienced academic orthodontists from northern Poland and five colleagues from Chile to refine the questions in terms of content as well as language. The questionnaire consisted of 25 questions and was designed using Google Forms (Google, Mountain View, CA, USA) tool. The questionnaire was delivered to the target group by publication in six social (three in Poland and three in Chile) media groups of 52,642 members. The participants were recruited in the Facebook groups for undergraduate students and graduates of humanistic universities.

The survey was anonymous; however, the Forms Tool was adjusted to require prior Google account verification in order to prevent multiple completion of the survey or repeated delivery of the same questionnaire. The post containing the link to the questionnaire was removed from the social media as soon as the study group was completed.

The questions were as follows:

1. How old are you?

- 18–21
- 22–25
- 25–30

2. Gender

- Male
- Female
- Other

3. What is the population of Your city?

- Above 500 thousand inhabitants
- Between 100 and 500 thousand inhabitants
- Between 50 thousand and 100 thousand inhabitants
- Below 50 thousand inhabitants

4. Are you planning to have an orthodontic treatment?

- No
- I don’t know
- Yes, in 6 months’ time
- Yes, in 12 months’ time
- Yes, in three years’ time
- Yes, in five years’ time

5. What kind of appliance are You interested in? (Multiple choice question)

- Metal fixed appliance
- Esthetic ceramic appliance
- Transparent aligners
- As recommended by the doctor
- None
- I don’t know, I am not sure

6. Have you been treated with: (multiple choice question).

- Removable acrylic appliance
- Metal fixed appliance
- Aesthetic fixed appliance
- Aligners
- I have not been treated

7. How long ago did your orthodontic treatment end?

- Less than 3 years ago
- between 3 and 5 years ago
- between 5 and 10 years ago
- More than 10 years ago
- I have not received orthodontic treatment

8. How many of your family or close friends wear or have worn aligners?

- None
- One
- Two
- More than 2

9. Which of the following brands do you recognize? (multiple choice question)

- Invisalign
- Clear aligner
- Dr. Smile
- Wizz
- Suresmile
- Alineadent
- Spark
- None

10. Where did you hear about aligners? (Multiple choice question)

- TV commercials
- Press
- Facebook
- Instagram
- TikTok
- Twitter
- From a general dentist
- From my orthodontist
- From colleagues and relatives
- None of the above
- I have never heard of aligners before

11. Can you imagine accepting orthodontic treatment without contacting an orthodontist (based on scans and photos taken by non-medical staff) if it would reduce the price of treatment?

- No
- I don’t know
- Yes

12. Do you find it acceptable if the progress of your treatment would not be controlled by a doctor?

- Yes
- No
- I don’t know

13. Is an orthodontic consultation by a general dentist instead of a specialist in orthodontics acceptable to you?

- Yes
- No
- I don’t know

14. Do you think orthodontic treatment is painful?

- Yes
- No
- I don’t know

15. What type of treatment do you think is less painful?

- Aligner treatment
- Treatment with fixed appliance
- Treatment with acrylic removable appliance
- Type of treatment has no effect on pain
- I don’t know

16. What features of orthodontic treatment are most important to you? (Multiple choice question)

- Acceptable price
- Esthetics of the appliance
- Invisibility of the appliance
- Treatment time
- Expected excellent result
- Partial improvement in tooth alignment
- Virtual prognosis and visualization of the treatment result
- No need for cooperation in wearing, changing appliance
- No dietary restrictions
- No speech disorders
- No irritation of the oral mucosa
- Brand recognition
- Short treatment time
- No grinding (stripping) of teeth
- No pain

17. Do you think aligner treatment is:

- Cheaper than metal fixed appliance
- More expensive than aesthetic fixed appliance
- Cheaper than aesthetic fixed appliance, and more expensive than metal fixed appliance
- There is no difference in price between the different types of treatment
- I don’t know

18. Do you know how long during the day you should wear aligners?

- Only at night
- 12 h
- 14-16 h
- 22-23 h
- I don’t know

19. Do you think teenagers who still have some milk teeth can be treated with aligners?

- Yes
- No
- I don’t know

20. What is your opinion on the indications for aligner treatment?

- Any malocclusion can be treated with aligners.
- Only non-complex malocclusion can be treated with this method.
- I have no idea

21. Do you think your doctor can verify that you wear aligners for as long as indicated?

- Yes
- No
- I don’t know

22. What do you think about the possible results of aligner treatment?

- I don’t know
- They are perfect
- Treatment with aligners is not as accurate as with fixed braces
- You can get better results than with fixed braces treatment

23. Do you think that the time of treatment with aligners is:

- I have no idea
- Similar to this of the treatment with fixed braces
- Shorter than fixed treatment
- Longer than fixed treatment

24. How long should the doctor be responsible for the outcome of the treatment after it is completed?

- For 1 year
- For 3 years
- For 5 years
- Lifelong

25. Would you be willing to undertake treatment with fixed braces, with a few additional aligners at the end of treatment to perfectly align your teeth, even if You had to pay for it?

- Yes.
- No.
- Don’t know.

### Statistical analysis

Number of answers were calculated both for single- and multi-choice questions. Significance of the differences in the answer frequencies across states, ages and genders was assessed using *χ*^2^ test. Difference was considered significant at *p* < 0.05. The *R* statistical program, ver.4.2.2 (The R Foundation for Statistical Computing, Wirtschaftsuniversität Wien, Vienna, Austria) was used for the calculations.

## Results

The response rate was 1,99%, what stands for 1092 responses.

### Demographic characteristics of the respondents

#### Age

Whereas the number of the respondents in-between 18 and 21 and 22–25 age groups are comparable, the Chilean group of the respondents was larger. All three age groups were comparably represented. The difference between all the three age subgroups was statistically significant. In Chile, the largest subgroup was 26–30, which was in contrary the smallest in Poland. The other two groups in Chile represent a similar percentage of the total sample, while in Poland the largest group was 22–25 and 18–21 was considerably smaller (Table 1).

**Table 1:** Number of the respondents in each age group

Country/Age	18–21	22–25	26–30
Chile	181 (27%)	180 (26,9%)	309 (46,1%)
Poland	172 (40,8%)	205 (48,6%)	45 (10,6%)
Total	353 (32,3%)	385 (35,3%)	354 (32,4%)
Difference CL/PL	< 0.001	< 0.001	< 0.001

#### Gender

Other stands for non-binary gender types. In both countries, women made up the majority in the survey sample, but in Chile the difference between the number of men and women was more pronounced (Table 2).

**Table 2:** Gender-based composition of the study sample

Country/Gender	Female	Male	Other
Chile	448 (66,9%)	222 (30,1%)	0 (0%)
Poland	247 (58,3%)	171 (40,5%)	4 (1,2%)
Total	695	389	4
Difference CL/PL	0.004	0.011	0.045

#### Place of residence referring to number of inhabitants

**Fig. 1:**
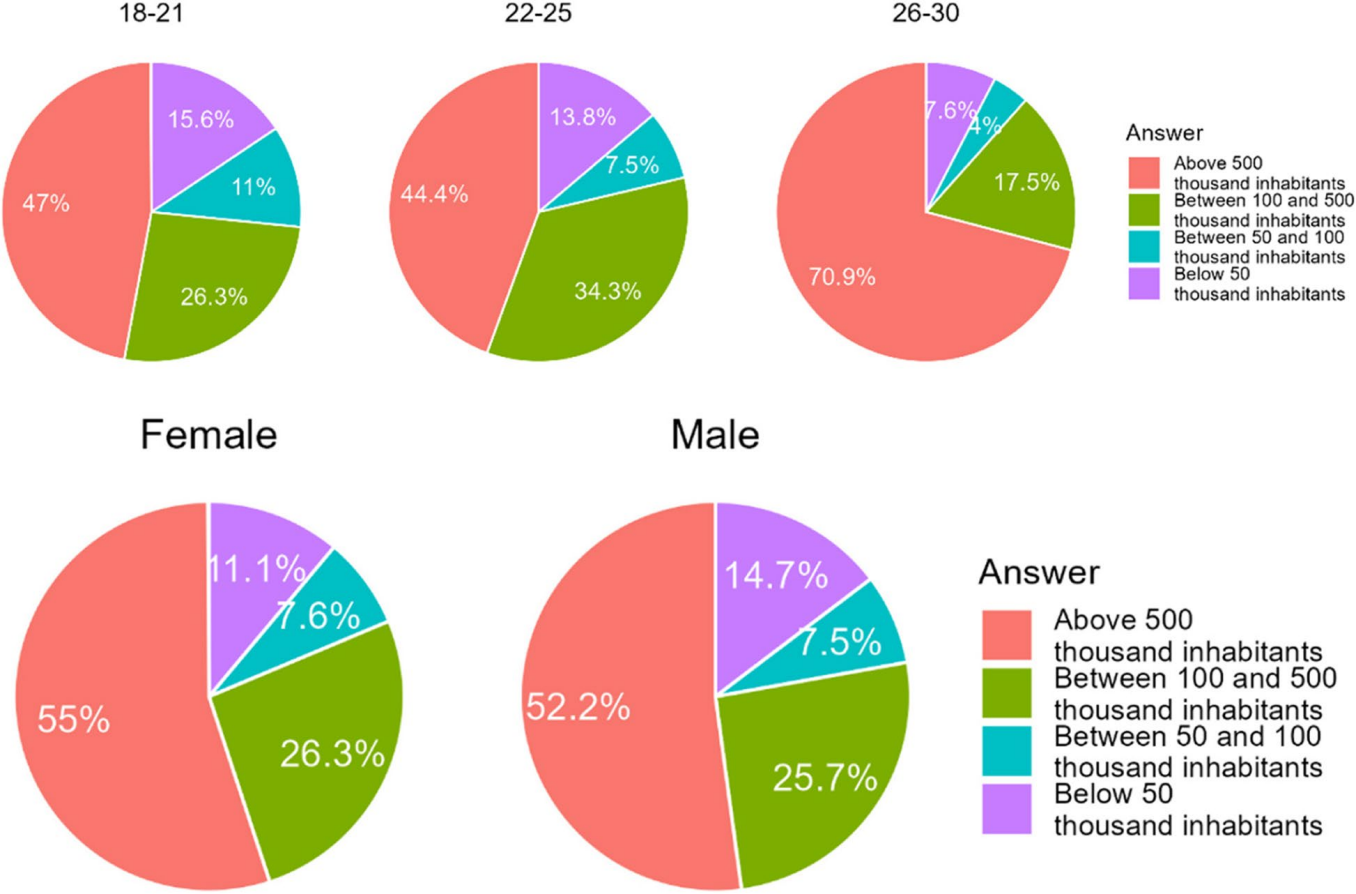
Age and gender of respondents in each cities of various population

The percentage of respondents from large cities (answer A, 500 K+) was significantly higher in Chile (*p* < 0.001) (Table 3). In Poland there were significantly more respondents living in smaller cities (*p* = 0.001 or less). However, the gender distribution of respondents was similar (Fig. 1).

**Table 3:** Number of respondents divided according to the population of the city of residence

Country/City population	> 500 k (A)	100 k–500 k (B)	50 k–100 k (C)	< 50 k (D)
Chile	531 (79,2%)	70 (10,5%)	36 (5,4%)	33 (4,9%)
Poland	57 (13,5%)	217 (51,4%)	46 (10,9%)	102 (24,2%)
Total	588 (52,8%)	287 (26,3%)	82 (19,5%)	135 (12,4%)
Difference CL/PL	< 0.001	< 0.001	0.001	< 0.001

### The orthodontic treatment demand in young adult population (between 18 to 30 years of age) in Poland and Chile

#### Do you have any plans to have an orthodontic treatment in upcoming future?

**Fig. 2:**
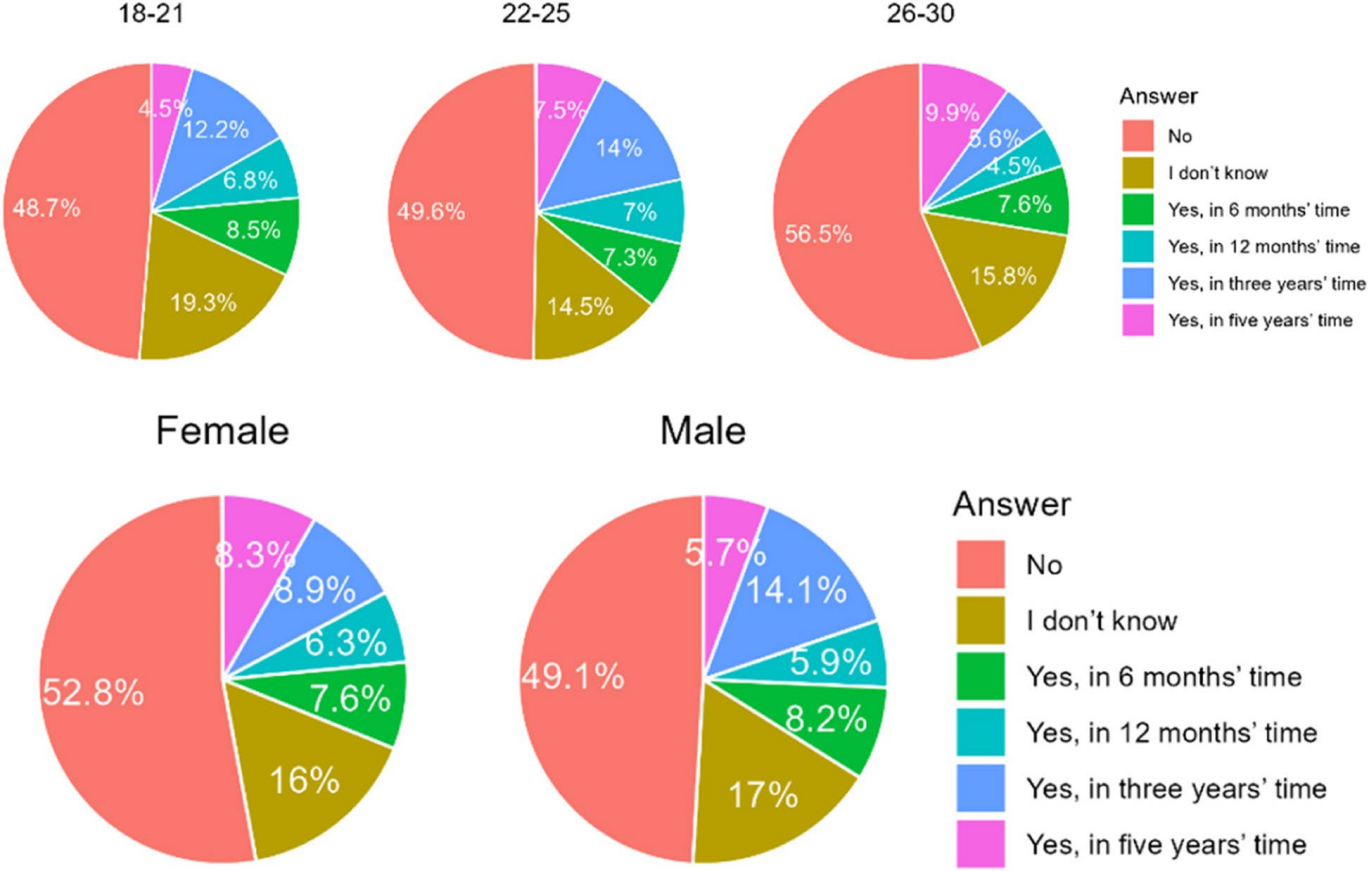
Age and gender of respondents willing to have orthodontic treatment

The majority of respondents do not want orthodontic treatment (51,6%) or do not know whether to start (16,5%) orthodontic treatment. Significantly more Poles than Chileans planned to begin orthodontic treatment. However, of those interested, Chileans would do sooner Poles - they would do in the long term (several years) (Table 4). A similar outlier of men and women are interested in orthodontic treatment, but men also are likely to do it rather in the next few years, then in the near future (Fig. 2).

**Table 4:** Orthodontic treatment demand of the respondents in upcoming time

Country	A. No	B. I don’t know	C. Yes, in 12 months time	D. Yes in 3 years time	E. Yes, in 5 years time	F. Yes, in 6 months time
Chile	398 (59,4%)	122 (18,2%)	38 (5,6%)	24 (3,6%)	17 (2,5%)	71 (10,6%)
Poland	165 (39,1%)	58 (13,7%)	47 (11,1%)	43 (10,9%)	100 (23,7%)	9 (2,1%)
Total	563 (51,6%)	180 (16,4%)	85 (7,8%)	67 (6,5%)	117 (10,7%)	80 (7,6%)
Difference CL/PL	< 0.001	0.064	0.002	< 0.001	< 0.001	< 0.001

#### What kind of orthodontic appliance would you choose?

The biggest share of the respondents in both countries did not show any interest in orthodontic treatment. From the ones interested in future orthodontic treatment a high percentage would follow the doctor’s recommendation in choosing the orthodontic appliance and was not sure about the type of orthodontic treatment. The largest per of those determined on the type of treatment before starting treatment (more than 10% of all respondents) in both countries, would be willing to be treated with aligners. Among Chileans, significantly more respondents declared willingness to be treated with aligners, while among Poles more declared willingness to be treated with standard braces (Table 5). Women were more likely than men to be interested in having their appliance selected by an orthodontist.

**Table 5:** Number of respondents intereseted in treatment with given orthodontic appliance

Country/Answer	A. Metal fixed appliance	B. Esthetic ceramic appliance	C. Transparent aligners	D. As recommended by the doctor	E. None	F. I don’t know, I am not sure
Chile	38 (5,7%)	17 (2,5%)	112 (16,7%)	121 (18,0%)	312 (46,6%)	134 (20,0%)
Poland	60 (14,2%)	27 (6,3%)	49 (11,6%)	104 (24,6%)	178 (42,2%)	55 (13,0%)
Total	98 (9,0%)	44 (4,0%)	161 (14,7%)	225 (20,6%)	490 (44,9%)	189 (17,3%)
Difference CL/PL	< 0.001	0.003	0.026	0.011	0.175	0.004

**Fig. 3:**
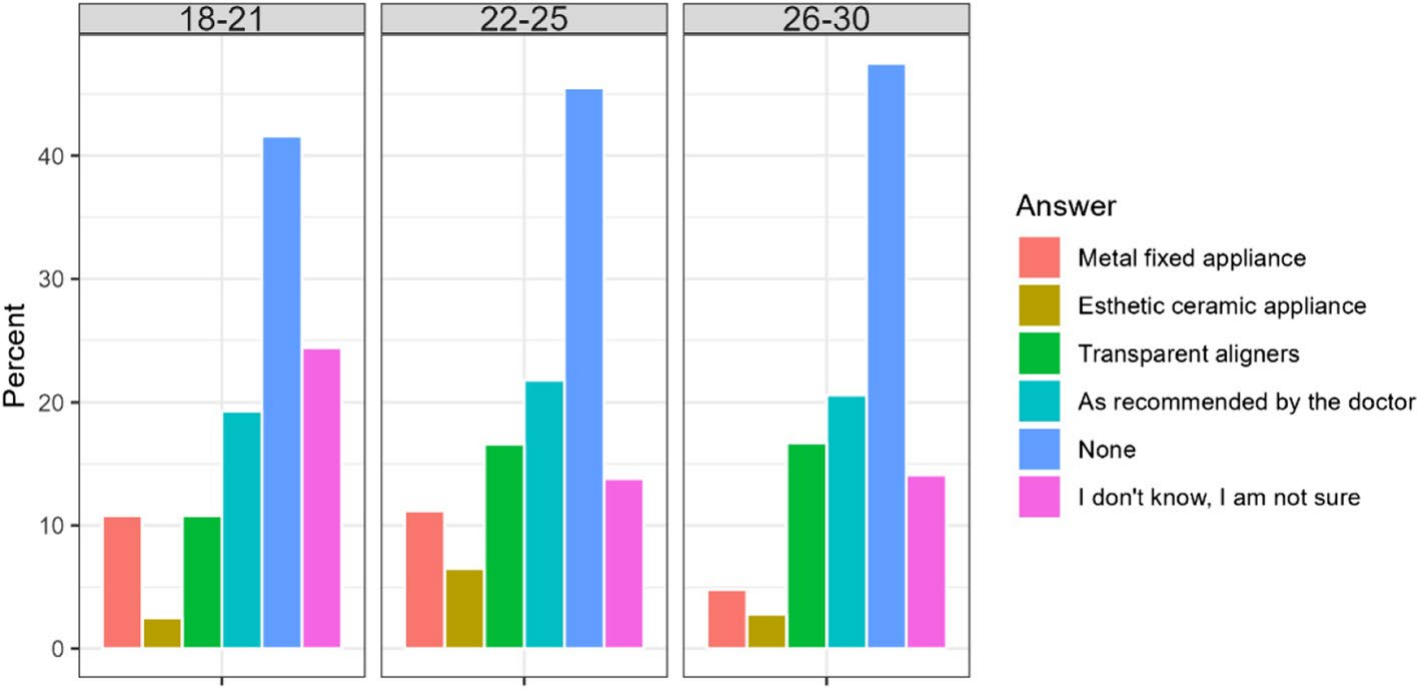
Percentage of respondents according to preferable type of treatment

It is worth to underline that patients older than 25 years either do not want orthodontic treatment or prefer transparent aligners to fixed appliance (Fig. 3).

#### Can you imagine accepting orthodontic treatment without contacting an orthodontist (based on scans and photos taken by non-medical staff) if it would lower the price of treatment?

**Fig. 4:**
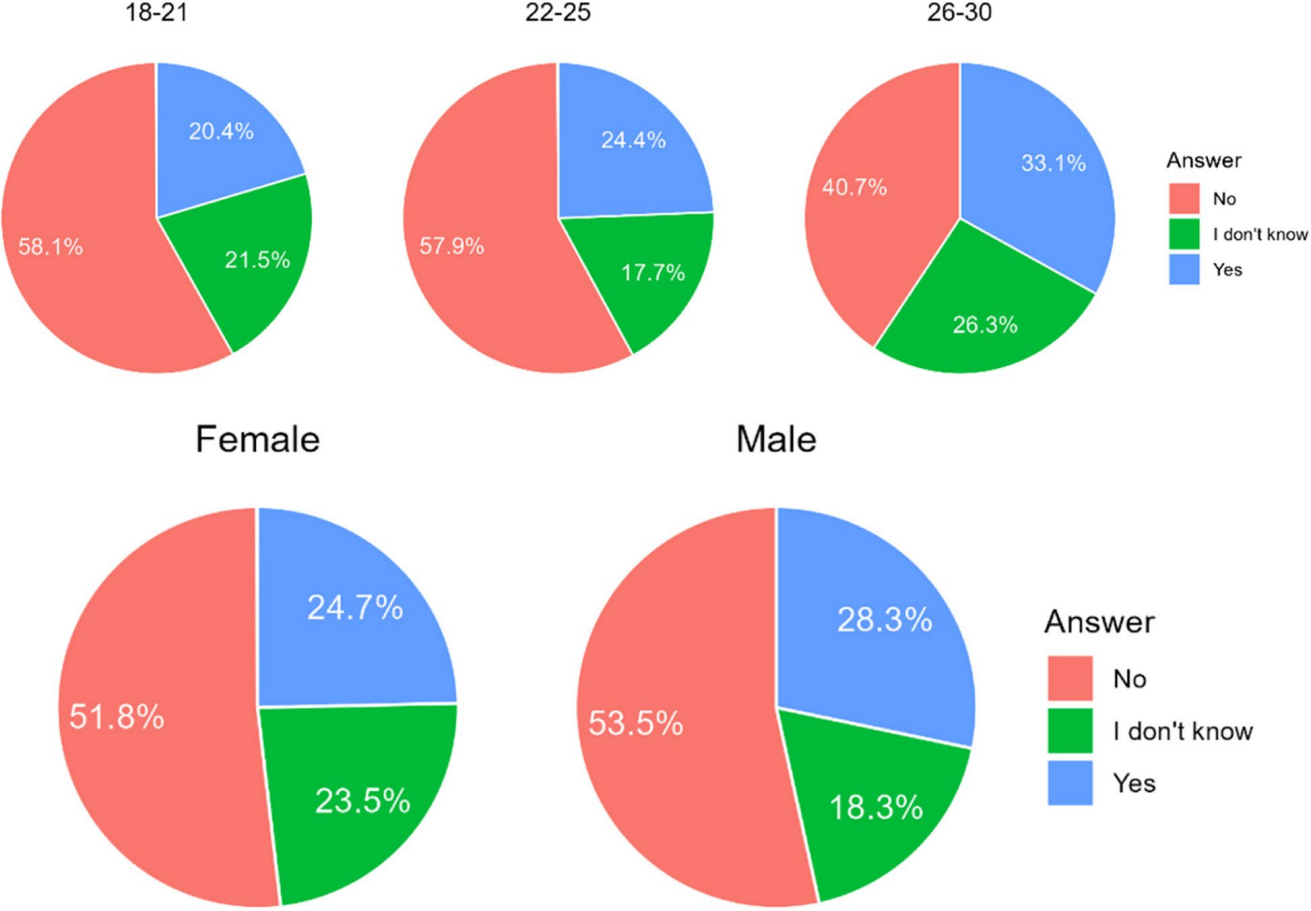
Percentage of respondents who answered it was acceptable for them to accepts a orthodontic treatment without being consulted by doctor

The majority of Poles would not agree to have an orthodontic treatment that was not planned by a doctor. Referring to Chile, opinions are divided. Interestingly, younger age groups were far more opposed to having treatment not planned by a doctor than the oldest age group (Table 6). There were no significant correlations related to gender (Fig. 4).

**Table 6:** Answers on acceptability of an orthodontic treatment in non-medical condition

Country	A. No	B. I don’t’ know	C. Yes
Chile	266 (39,7%)	184 (27,5%)	220 (32,8%)
Poland	306 (72,5%)	53 (12,6%)	63 (14,9%)
Total	572 (52,3%)	237 (21,7%)	283 (25,9%)
Difference CL/PL	< 0.001	< 0.001	< 0.001

#### Do you find it acceptable that the progress of your treatment is not controlled by a doctor?

**Fig. 5:**
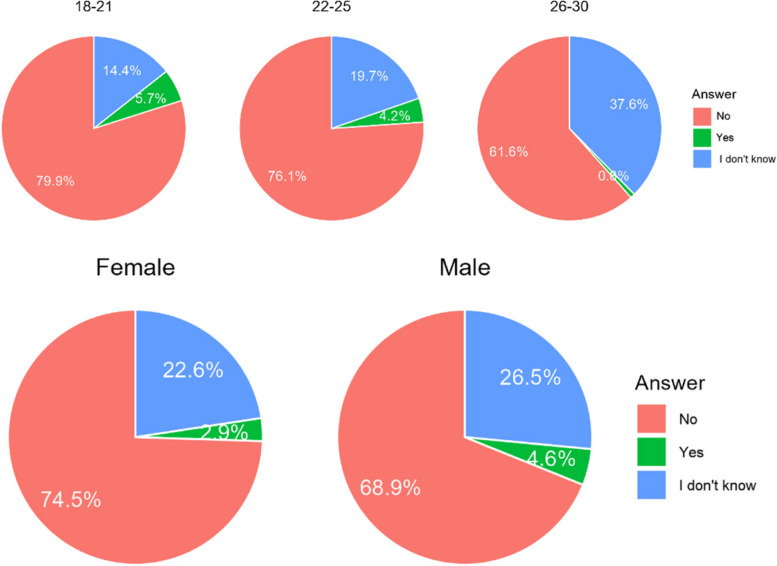
Answers of respondents on question whether they found acceptable to have a treatment which would not be controlled by the clinician

In both countries most of the respondents (85% in Poland and 65% of Chileans) found it unacceptable that the treatment would not be controlled by a doctor (Table 7). However, there is a much bigger group of the future patients, that could consider treatment without supervision of a doctor. Younger age groups are far more opposed to having treatment not supervised by dentist than the oldest age group (Fig. 5).

**Table 7:** Answers of respondents on question whether they found acceptable to have a treatment which would not be controlled by the clinician

Country	A. No	B. I don’t’ know	C. Yes
Chile	434 (64,8%)	0 (0%)	236 (35,2%)
Poland	359 (85,1%)	39 (9,2%)	24 (5,7%)
Total	793 (67,7%)	39 (3,6%)	260 (23,7%)
Difference CL/PL	< 0.001	< 0.001	< 0.001

#### Is an orthodontic consultation by a general practitioner dentist instead of a specialist in orthodontic acceptable to you?

**Fig. 6:**
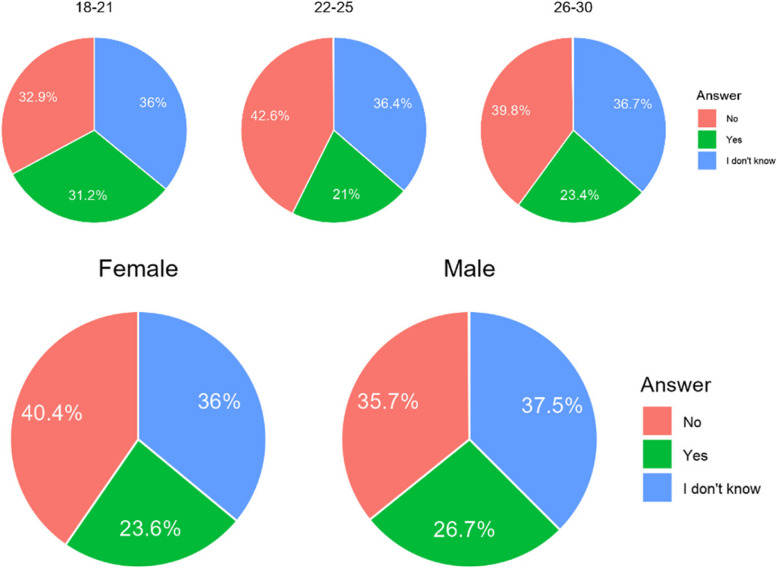
Share of respondents, who found acceptable to be treated by general practitioner instead of specialist in orthodontics

In both countries, it makes no difference whether the treatment would be consulted by a general practitioner instead of an orthodontic specialist. Opinions are sharply divided (Table 8). Respondents in the middle and upper age bracket are far more attentive to the title of specialist than younger respondents (Fig. 6).

**Table 8:** Share of respondents, who found acceptable to be treated by general practitioner instead of specialist in orthodontics

Country	A. No	B. I don’t know	C. Yes
Chile	256 (38,2%)	181 (27,0%)	233 (34,8%)
Poland	165 (39,1%)	93 (22,0%)	164 (38,9%)
Total	421 (38,6%)	274 (25,1%)	397 (36,4%)
Difference CL/PL	0.818	0.076	0.193

#### What features of orthodontic treatment are most important to you? (multiple choice question)

**Fig. 7:**
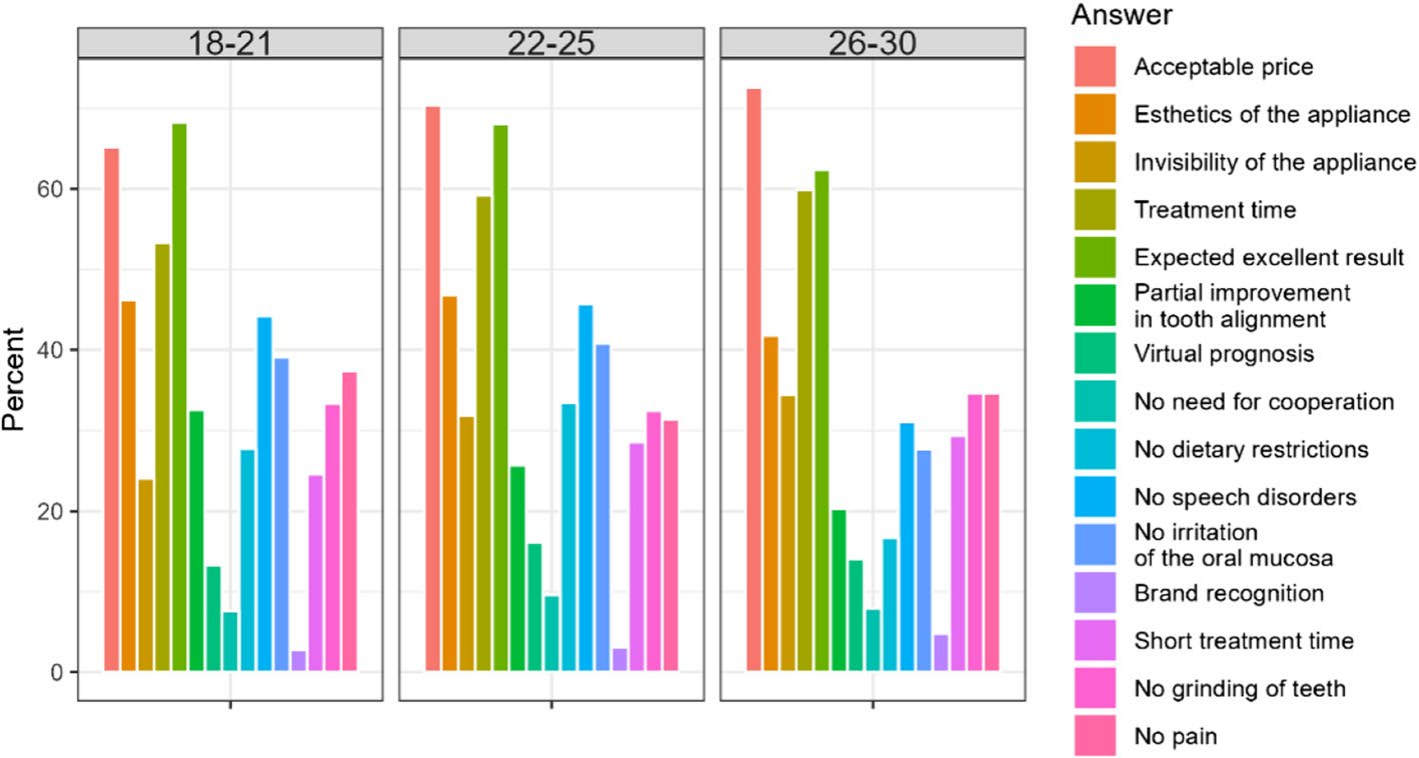
Differences in most important characteristics according to age, country and gender of the respondents

Among the most important characteristics named in both countries are: acceptable price, aesthetics of the appliance, treatment time, expected excellent result, no speech disorders and no irritation of oral mucosa. The latter characteristics can be considered of lower importance. The differences between the countries are made bold in Table 15. For Polish respondents, the most important aspects were convenience and ideal treatment outcome, while for Chileans the short treatment time or lack of stripping (Table 9). Female respondents significantly more often than men indicated acceptable price, aesthetics of the appliance, invisibility of the appliance and treatment time. Younger patients (18–21) were not as demanding regarding final treatment result as respondents in older groups. However, they found more important no speech disorders and no irritation of the oral mucosa more important than other age groups (Fig. 7).

**Table 9:** Characteristics of treatment considered most important by the respondents (significant differences in bold)

**Country/Answer**	A. Acceptable price	B. Aesthetics of the appliance	**C. Invisibility of the appliance**	**D. Treatment time**	**E. Expected excellent result**
Chile	475 (70,9%)	311 (46,4%)	230 (34,3%)	404 (60,3%)	419 (62,5%)
Poland	283 (67,1%)	180 (42,7%)	100 (23,7%)	224 (53,1%)	305 (72,3%)
Sum	758 (69,4%)	491 (45,0%)	330 (30,2%)	628 (57,5%)	724 (66,3%)
Difference CL/PL	0.204	0.248	< 0.001	0.022	0.001
**Country/Answer**	**F. Partial improvement in tooth alignment**	G. Virtual prognosis and visualization of the treatment result	H. No need for cooperation in wearing, changing appliance	**I. No dietary restrictions**	**J. No speech disorders**
Chile	201 (30%)	97 (14,5%)	64 (9,6%)	121 (18,1%)	233 (34,8%)
Poland	85 (20,1%)	62 (14,7%)	28 (6,6%)	165 (39,0%)	209 (49,5%)
Sum	286 (26,2%)	159 (14,6%)	92 (8,4%)	286 (26,2%)	442 (38,6%)
Difference CL/PL	< 0.001	0.992	0.115	< 0.001	< 0.001
**Country/Answer**	***K. no*** **irritation of the oral mucosa**	L. Brand recognition	M. Short treatment time	**N. No grinding (stripping) of teeth**	O. No pain
Chile	202 (30,1%)	32 (4,8%)	190 (28,4%)	256 (38,2%)	219 (32,7%)
Poland	191 (45,3%)	7 (1,7%)	111 (26,3%)	110 (26,1%)	157 (37,2%)
Sum	393 (36,0%)	39 (36%)	301 (27,6%)	366 (33,5%)	376 (34,4%)
Difference CL/PL	< 0.001	0.011	0.503	< 0.001	0.143

#### How long should the doctor be responsible for the outcome of the treatment after it is completed?

**Fig. 8:**
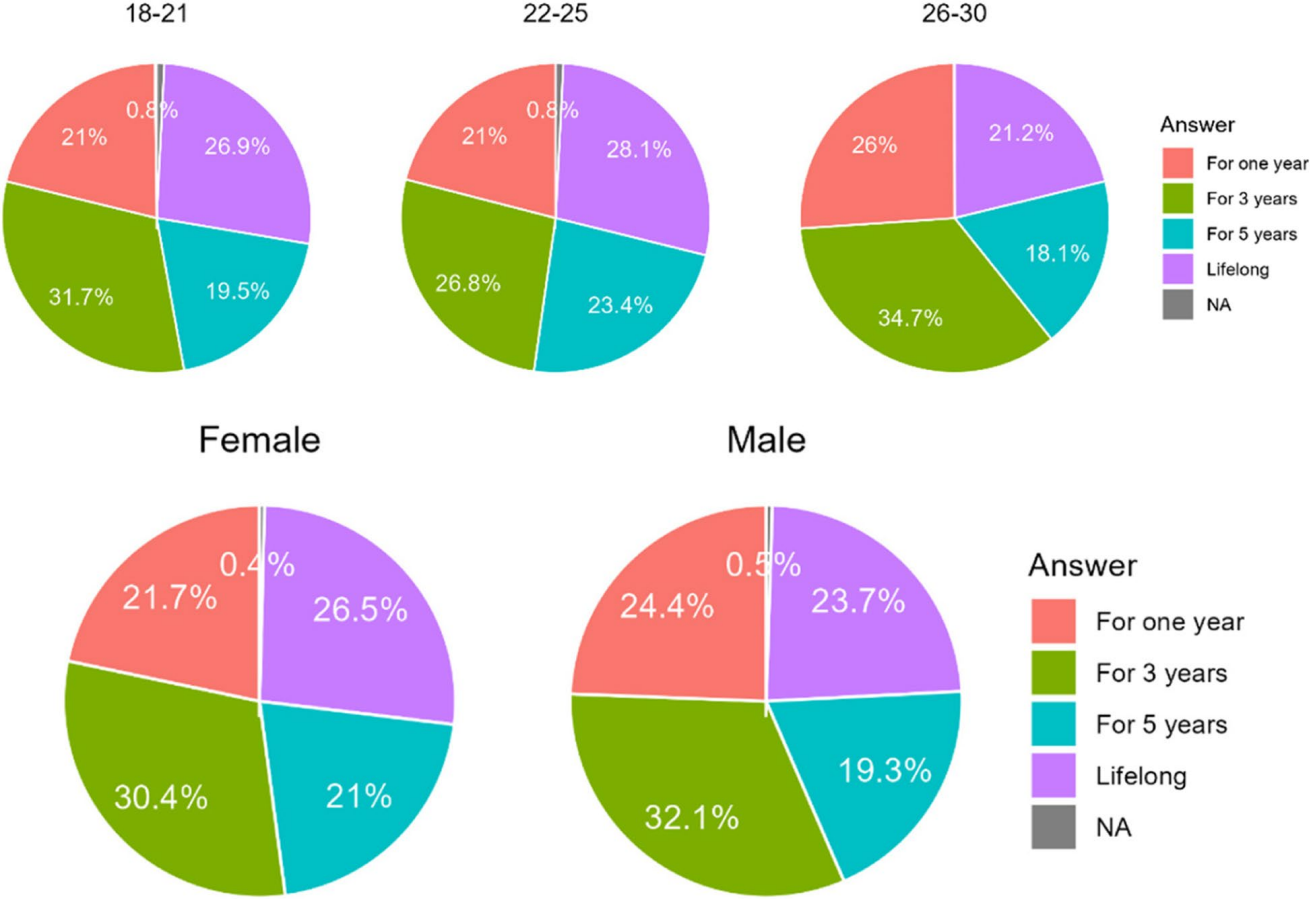
Differences in answers according to age, country and gender of the respondents

While Chileans chose much more often shorter period of doctor’s responsibility, Polish tent to choose longer periods of time (Table 10). Females also chose longer periods of time then males. N/A stated for no response (Fig. 8).

**Table 10:** Insights of respondents to amount of time in which doctor should be responsible for outcome of treatment

Country/Answer	A. 1 year	B. 3 years	C. 5 years	D. Whole life
Chile	186 (27,8%)	217 (32,4%)	101 (15,0%)	166 (24,8%)
Poland	61(14,5%)	121(28,9%)	122 (28,9%)	116 (26,5%)
Sum	247 (22,6%)	338 (31,0%)	223 (20,4%)	282 (26.0%)
Difference CL/PL	< 0.001	0.282	< 0.001	0.474

#### Would you be willing to have treatment with fixed braces, with a few additional aligners at the end of treatment to perfectly align your teeth, even if you had to pay for it?

**Fig. 9:**
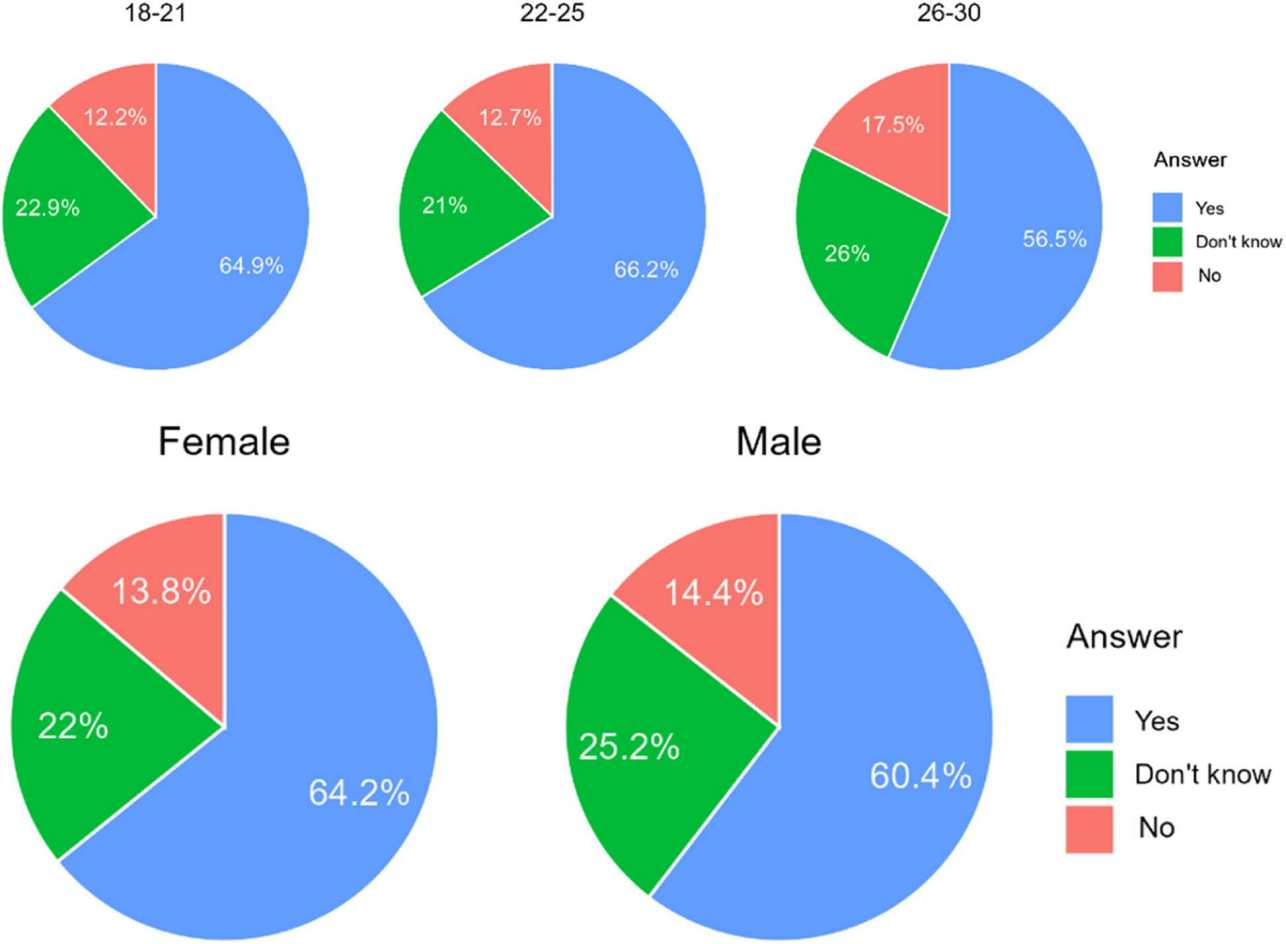
Differences in answers according to age and gender of the respondents

Most respondents from both countries would be keen on paying more in order to get perfect alignment of the teeth. However, Chileans would do significantly less frequently than Poles (Table 11). Younger respondents and females would do much more probably than older respondents and males (Fig. 9).

**Table 11:** Percentage of respondents willing to pay extra for aligners to achieve perfect alignment of teeth

Country/Answer	A. Yes	B. I don’t know	C. No
Chile	405 (60,4%)	152 (22,7%)	113 (16,9%)
Poland	279 (66,1%)	102 (24,2%)	41 (9,7%)
Sum	684 (62,6%)	254 (23,2%)	154 (14,2%)
Difference CL/PL	0.069	0.623	0.001

### Previous orthodontic experience of the young adults

#### Experience in completed orthodontic treatment

**Fig. 10:**
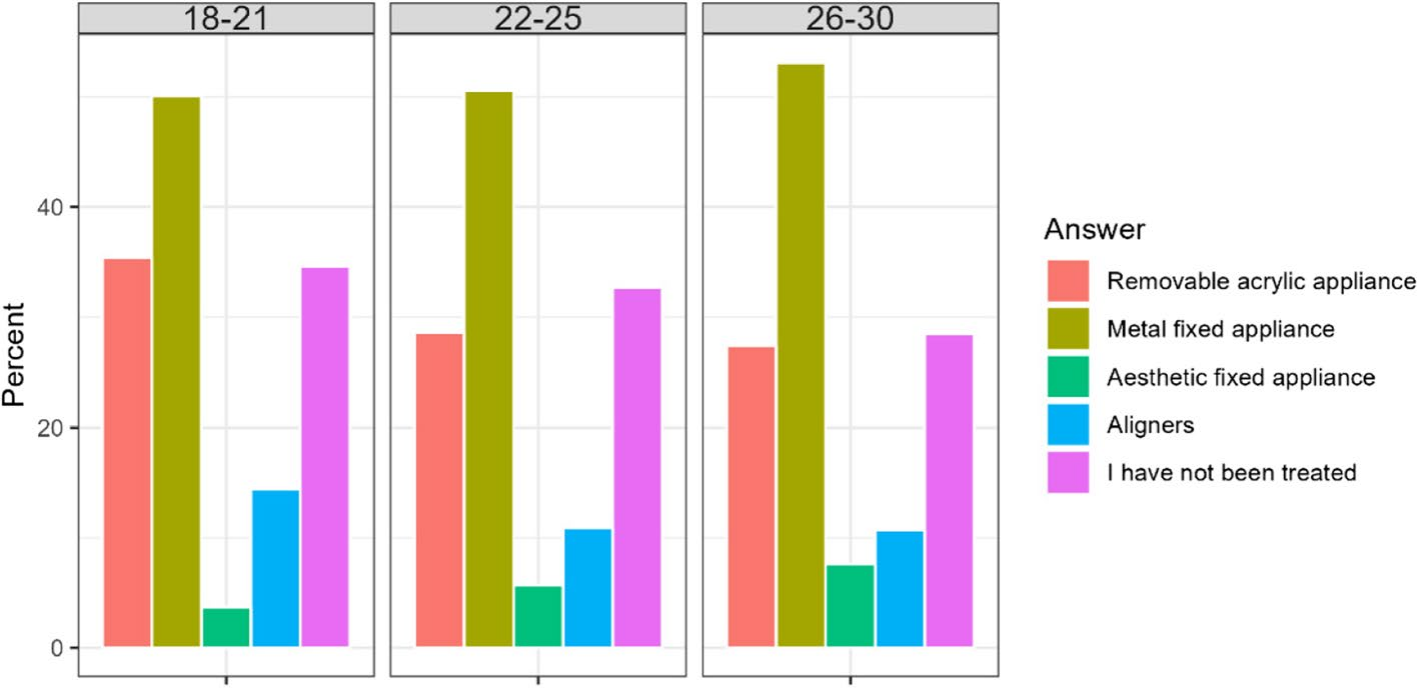
Percentage of respondents according to their experience with orthodontic treatment

Most respondents have already had experience with orthodontic treatment. Proportionally more Poles have had treatment with removable appliances, while for fixed braces and aligners this percentage can be considered similar. Proportionally more Poles than Chileans have had no contact with an orthodontist (Table 12). Younger respondents were more often treated with removable appliances than older respondents. Women were statistically more likely to be treated with aligners. Older patients were not treated more frequently (Fig. 10).

**Table 12:** Answers of the participants about previous type, if any, of orthodontic treatment

Country/Answer	A. Removable acrylic appliance	B. Metal fixed appliance	C. Esthetic fixed appliance	D. Aligners	E.I have not been treated
Chile	196 (29,2%)	426 (63,6%)	45 (6,7%)	99 (14,8%)	168 (25,0%)
Poland	136 (32,2%)	134 (31,8%)	17 (4,0%)	32 (7,6%)	181 (42,9%)
Total	332 (30,4%)	560 (51,2%)	62 (5,7%)	131 (12,0%)	349 (32,0%)
Difference CL/PL	0.331	< 0.001	0.083	< 0.001	< 0.001

#### How long ago did the respondents undergo an orthodontic treatment?

**Fig. 11:**
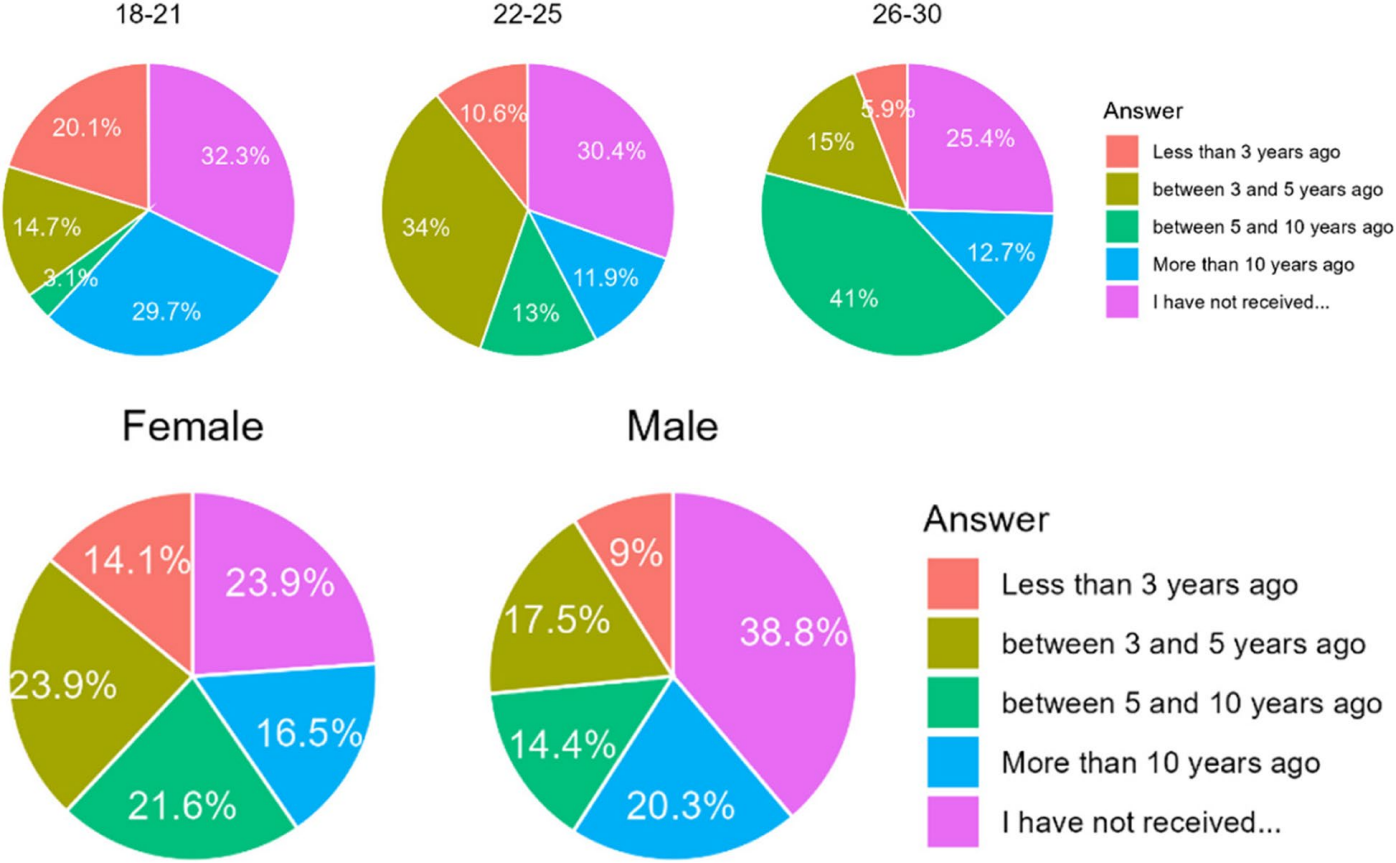
Age, gender and country of respondents according to the time they were treated

Proportionately, more Poles were treated with orthodontics more than 10 years ago, while Chileans dominate the groups where respondents claimed to be treated later. Statistically signifcatly more Poles and more males have never been treated orthodontically (Table 13). There are significant differences between age groups in the frequency with which all answers were selected. However, no significant correlation or pattern can be found within this. On the other side, it is apparent that women were treated more often and that this had place significantly closer to the time of the study (teenage years) than was the case for men (Fig. 11).

**Table 13:** Number of respondents according to time when they undergo orthodontic treatment

Country/Answer	A. Less than 3 years ago	B. Between 3 to 5 years ago	C. Between 5 to 10 years ago	D. More than 10 years ago	E. I have not received orthodontic treatment
Chile	94 (14,1%)	148 (22,1%)	147 (21,9%)	137 (20,4%)	144 (21,5%)
Poland	39 (9,2%)	88 (20,9%)	59 (14,0%)	59 (14,0%)	177 (41,9%)
Sum	133 (12,2%)	236 (21,6%)	206 (18,9%)	196 (17,9%)	321 (29,4%)
Difference CL/PL	0.024	0.683	0.001	0.009	< 0.001

#### How many of your family or close friends wear or have worn aligners?

**Fig. 12:**
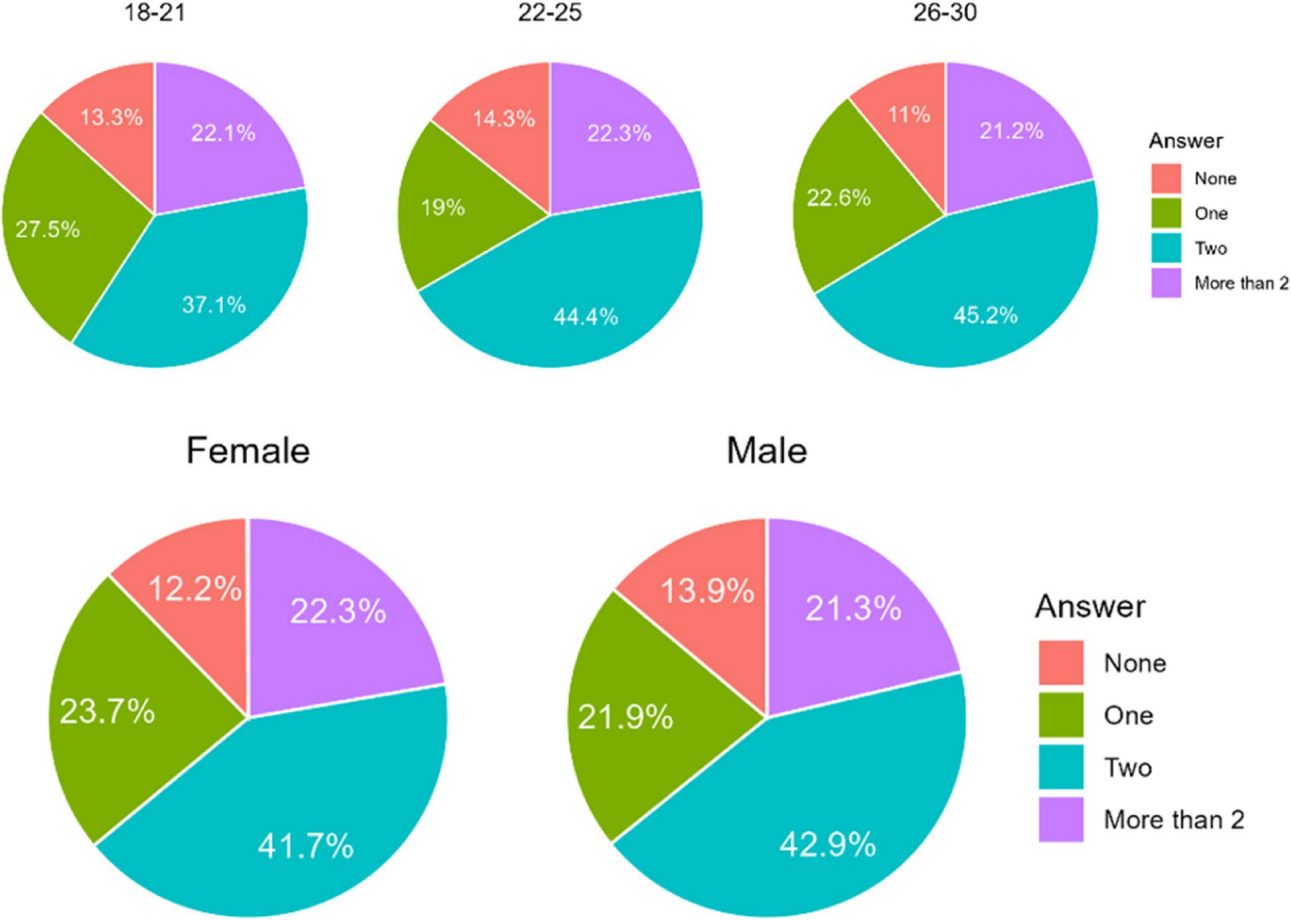
Age, country and gender of respondents according to number of their assosciates ever wearing aligners

The majority of respondents did not know anyone, who had worn aligners. Intrestingly, one fourth of the respondents knew more then two persons who had aligners (Table 14). Therefore, it may be supposed that it can be associated to social status. No significant correlations were found regarding age or gender (Fig. 12).

**Table 14:** Number of respondents according to number of their assosciates ever wearing aligners

Country/Answer	A. None	B. One	C. Two	D. More than two
Chile	277 (41,3%)	139 (20,7%)	91 (13,6%)	163 (24,4%)
Poland	185 (43,8%)	100 (23,7%)	50 (11,8%)	87 (20,6%)
Sum	462	239	141	250
Difference CL/PL	0.453	0.283	0.460	0.178

### The knowledge of young adults on fixed and aligner orthodontic treatment

#### Which of the following brands do you recognize?

**Fig. 13:**
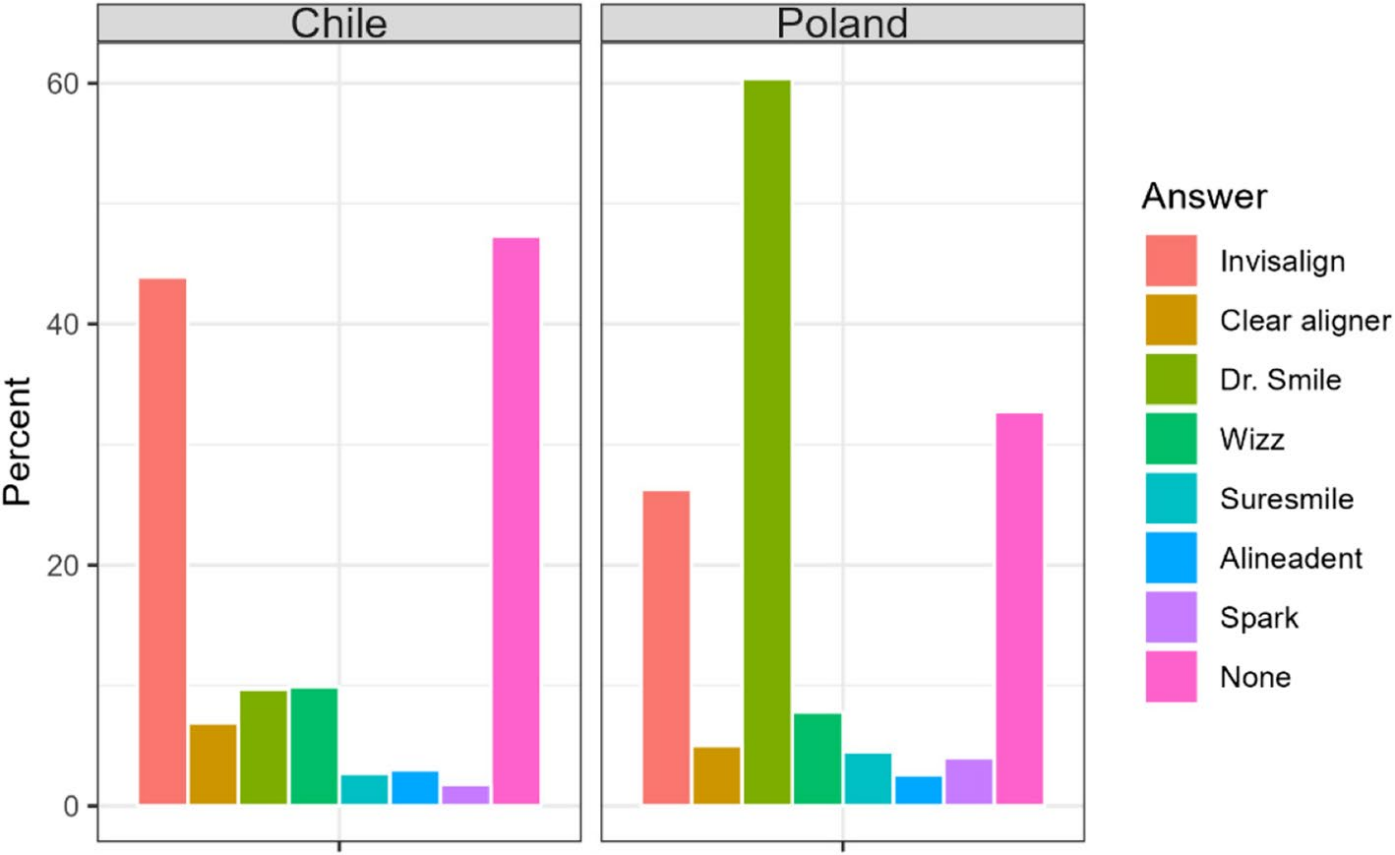
Knowledge of the brands in question among respondents

Almost a half of respondents did not know any aligner brand. However, about 38% of the respondents recognized Invisalign and about 30% recognized Dr. Smile, which means that these brands can be considered distinguishable. The other brands were not common among the respondents. In Chile the most distinguishable brand was Invisalign, whereas in Poland was Dr. Smile (Table 15, Fig. 13).

**Table 15:** Knowledge of the brands in question among respondents

Country/Answer	A. Invisalign	B. Clear aligner	C. Dr. Smile	D. Wizz	E. Suresmile	F. Alineadent	G. Spark	H. None
Chile	294	46	65	66	18	20	12	317
Poland	111	21	255	33	19	11	17	138
Sum	405	67	320	99	37	31	29	455
Difference	< 0.001	0.255	< 0.001	0.303	0.149	0.857	0.041	< 0.001

#### Medium providing information about aligners

**Fig. 14:**
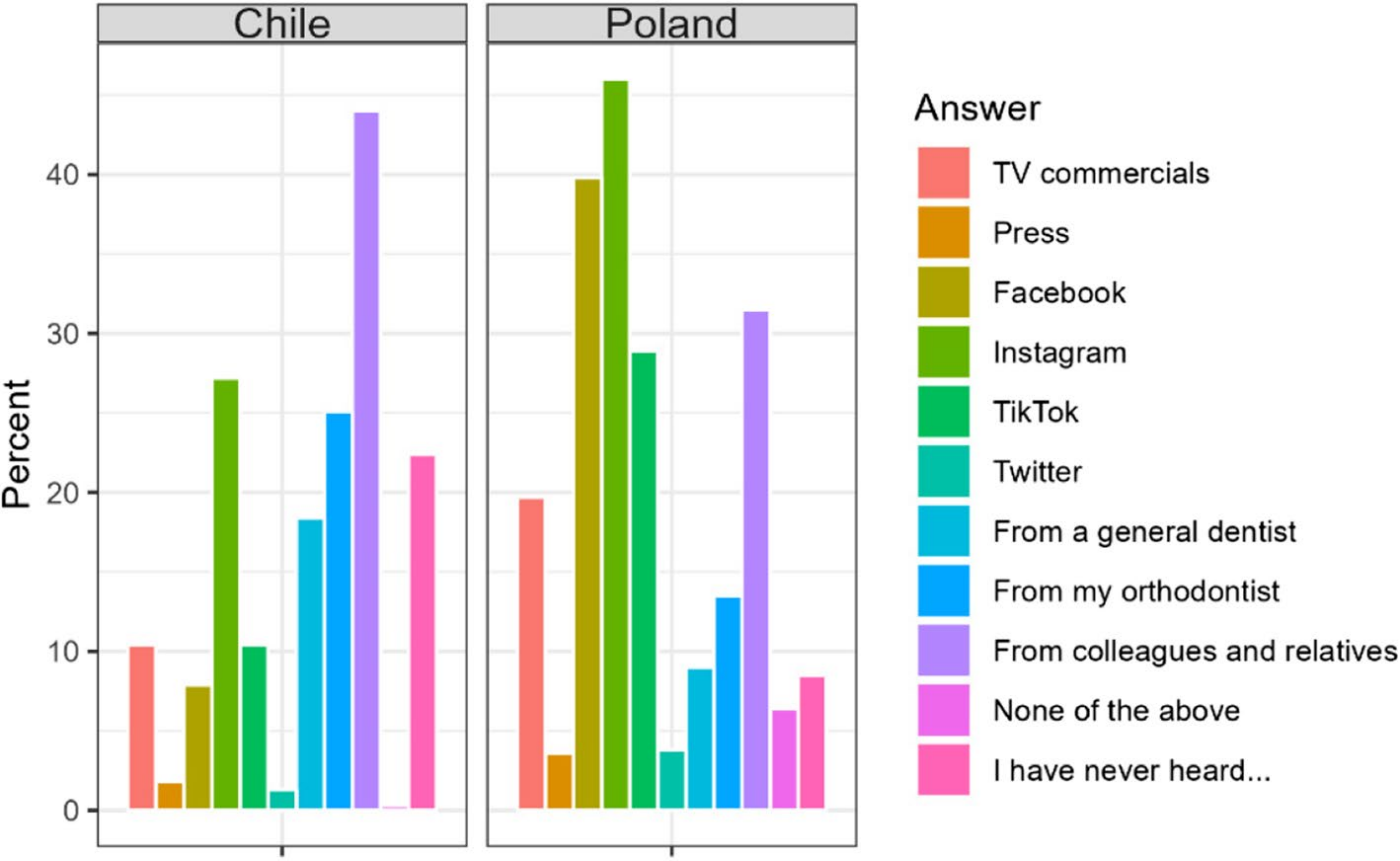
Source of information about the aligners among the respondents

In Chile the most important sources of information about aligners were orthodontist, dentists and associates. In contrary, in Poland, the most important source were social media of different kind (Table 16, Fig. 14).

**Table 16:** Source of information about the aligners among the respondents

Country	A. TV/Youtube	B. Press	C. Facebook	D. Instagram	E. Tiktok
Chile	70 (10,4%)	12 (1,8%)	53 (7,9%)	182 (27,1%)	70 (10,4%)
Poland	83 (19,7%)	15 (3,6%)	168 (39,8%)	194 (46,0%)	122 (28,9%)
Sum	153 (14,0%)	27 (2,5%)	221 (20,2%)	376 (34,4%)	192 (17,6%)
Difference CL/PL	< 0.001	0.104	< 0.001	< 0.001	< 0.001
F. Twitter	G. From my generally practitioner	H. From my orthodontist	I. From my colleagues	J. None	K. I have never heard about the aligners before
9 (1,3%)	123 (18,4%)	168 (25,1%)	295 (44,0%)	2 (0,2%)	150 (22,4%)
16 (3,8%)	38 (9,0%)	57 (13,5%)	133 (31,5%)	27 (6,4%)	36 (8,5%)
25 (2,3%)	161 (14,7%)	225 (20,6%)	428 (39,1%)	29 (2,6%)	186 (17,0%)
0.015	< 0.001	< 0.001	< 0.001	< 0.001	< 0.001

#### Do you think orthodontic treatment is painful?

**Fig. 15:**
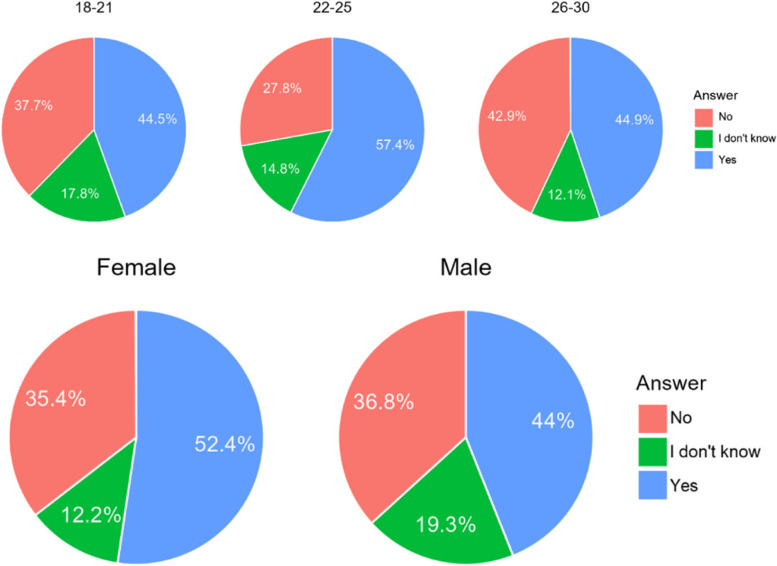
Share of the respondents according to age and gender

Most respondents hold orthodontic treatment for painful experience (Table 17). The respondents in age group 22–25 considered orthodontic treatment painful much more frequently than other age subgroups. Women were far more likely to admit that they found orthodontic treatment painful than men (Fig. 15).

**Table 17:** Answers on mentioned question

Country	A. No	B. I don’t know	C. Yes
Chile	260 (38,8%)	65 (9,7%)	345 (51,5%)
Poland	132 (31,3%)	98 (23,2%)	192 (45,5%)
Sum	392 (35,9%)	163 (14,9%)	537 (49,2%)
Difference CL/PL	0.014	< 0.001	0.062

#### What kind of treatment do you think is less painful?

**Fig. 16:**
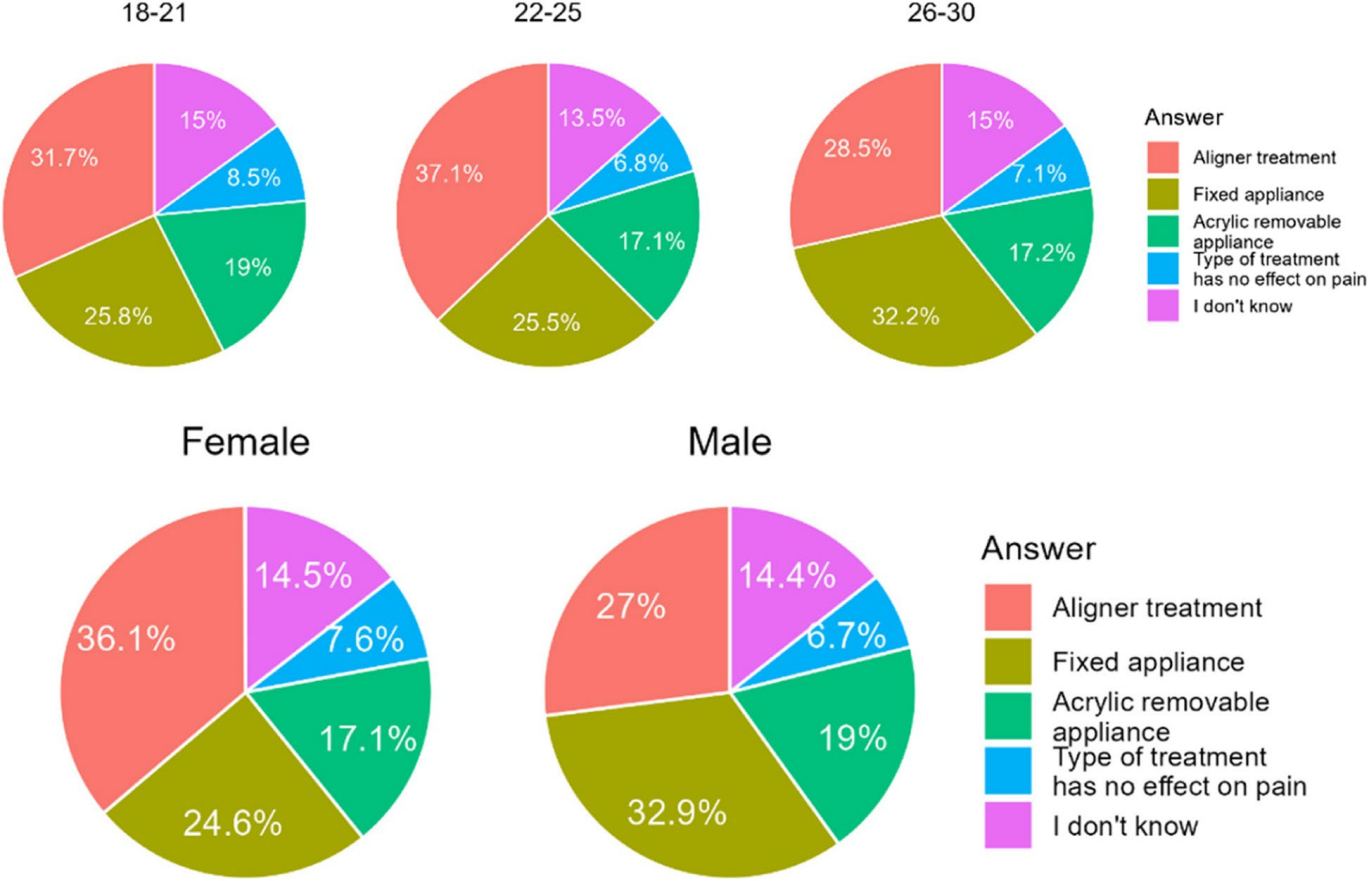
Least painful treatment type according to respondents

Patients primarily find treatment with aligners and acrylic removable appliances less painful than treatment with fixed braces (Table 18). Women and those aged 22–25 were also more likely to choose this answer (Fig. 16).

**Table 18:** Least painful treatment type according to respondents

Country	A. Aligner treatment	B. I don’t know	C. Treatment with acrylic removable appliance	D. Treatment with fixed appliance	E. Type of treatment has no effect on pain
Chile	200 (18,3%)	170 (15,6%)	151 (22,5%)	55 (8,2%)	91 (13,6%)
Poland	156 (37,0%)	133 (31,5%)	40 (9,5%)	26 (6,2%)	67 (15,8%)
Sum	356 (32,6%)	303 (27,7%)	193 (45,7%)	81 (19,2%)	158 (37,4%)
Difference CL/PL	0.017	0.032	< 0.001	0.255	0.336

#### Estimation of orthodontic treatment price

**Fig. 17:**
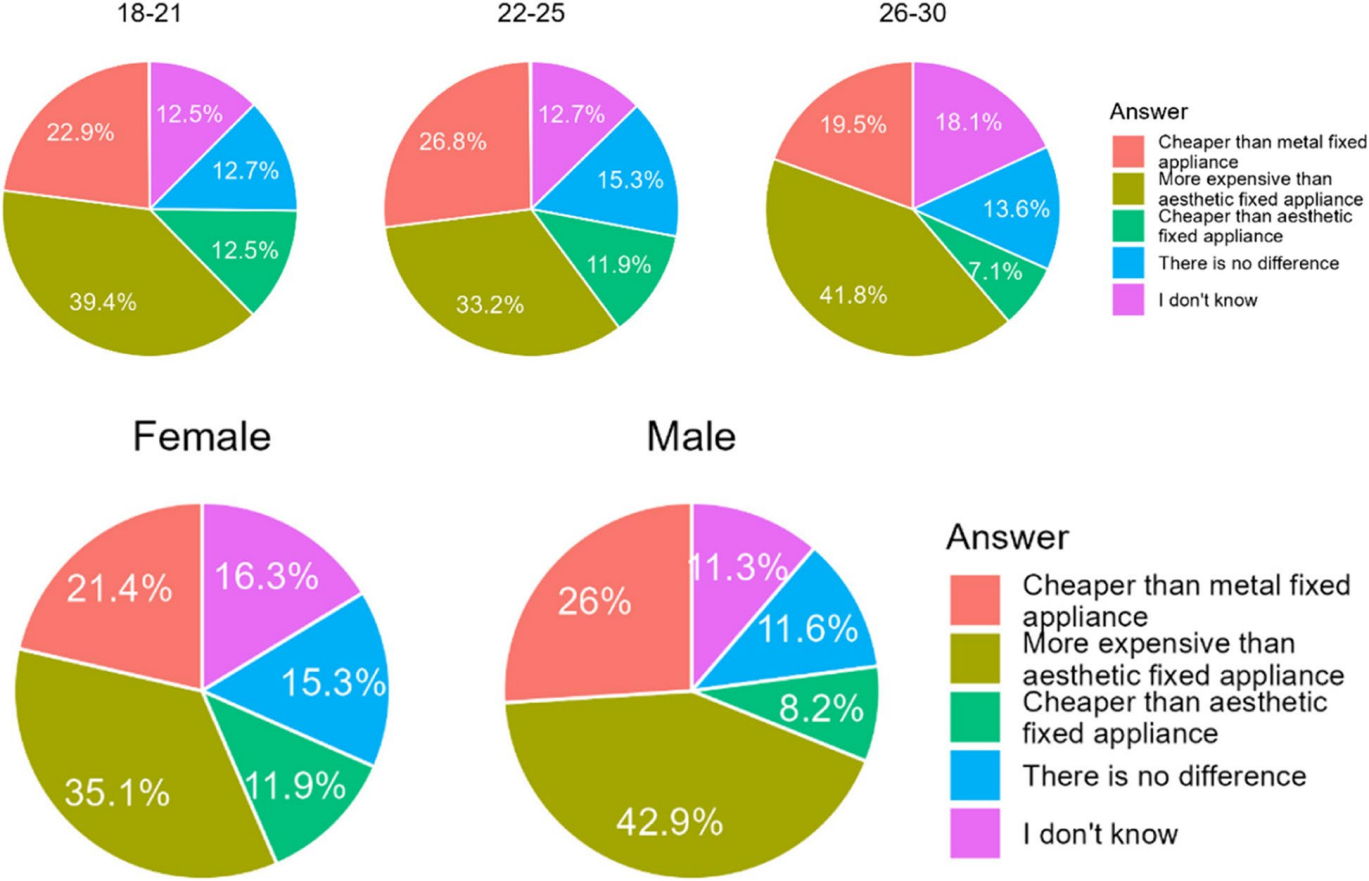
Source of information about aligners among the respondents

Many respondents hold that aligner treatment is most expensive of all three options of treatment. However, the majority of respondents chose other answers. Interestingly, more Poles than Chileans claimed that there was no difference in price (Table 19). Men were significantly more likely than women to indicate that aligner treatment was more expensive than fixed appliance treatment (Fig. 17).

**Table 19:** Estimation of orthodontic treatment price by the respondents

Country/Answer	A. Cheaper than metal fixed appliance	B. More expensive than aesthetic fixed appliance	C. Cheaper than aesthetic fixed appliance, and more expensive than metal fixed appliance	D. There is no difference in price between the different types of treatment	E. I don’t know
Chile	154 (23,0%)	230 (34,3%)	63 (9,4%)	26 (3,9%)	157 (23,4%)
Poland	99 (23,5%)	200 (29,9%)	52 (12,3%)	71 (16,8%)	0 (0%)
Sum	253 (23,2%)	430 (39,4%)	115 (10,5%)	97 (8,9%)	157 (14,4%)
Difference CL/PL	< 0.001	0.002	0.153	< 0.001	< 0.001

#### Do you know how long during the day you should wear aligners?

**Fig. 18:**
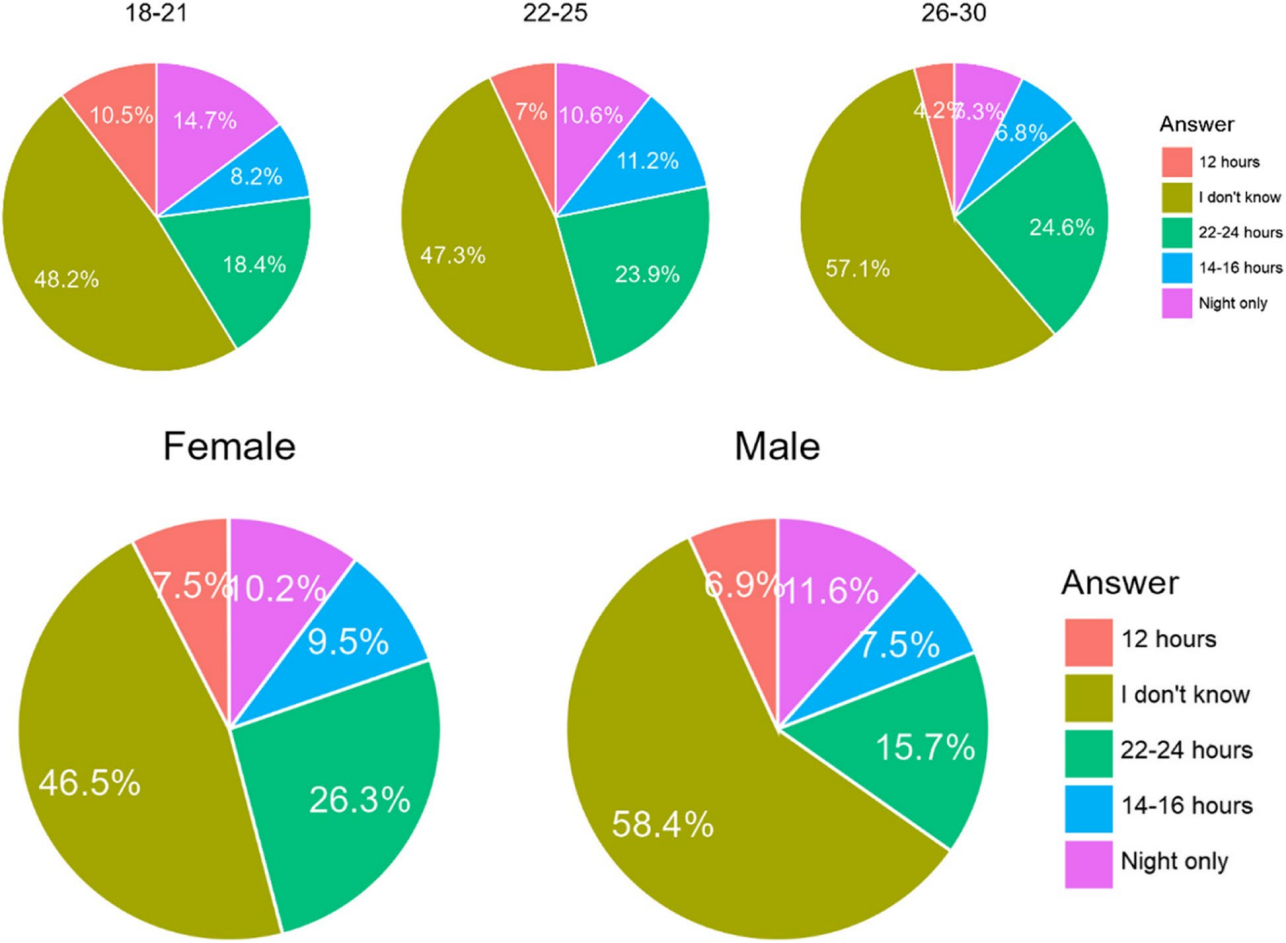
Share of the respondents according to age and gender

The majority of the respondents claimed that they don’t know or gave the wrong answer. Only about 23% of the respondents knew the correct answer. What is more, significantly more older respondents, more Chileans and more females knew the correct answer (Table 20). Older age subgroups knew the correct answer more frequently than the youngest one. However, significantly more female respondents knew how to wear aligners correctly (Fig. 18).

**Table 20:** Estimation of need to wear aligners on teeth by the respondents

Country/Answer	A. 12 hours	B. I don’t know	C. 22–24 hours	D. 14–16 hours	E. Only during the night
Chile	45 (6,7%)	316 (47,2%)	170 (25,4%)	60 (9,0%)	79 (11,7%)
Poland	34 (8,1%)	238 (56,4%)	74 (17,5%)	36 (8,5%)	40 (9,5%)
Sum	79 (7,2%)	554 (50,7%)	244 (22,3%)	96 (8,8%)	119 (10,9%)
Difference CL/PL	0.476	0.004	0.003	0.895	0.274

#### Do you think teenagers who still have milk teeth can be treated with aligners?

Only about 20% of the respondents knew the correct answer. There were no significant correlations regarding age groups or gender (Table 21).

**Table 21:** Answer to given question

Country/Answer	No	I don’t know	Yes
Chile	239 (35,7%)	301 (44,9%)	130 (19,4%)
Poland	118 (27,9%)	220 (52,4%)	84 (19,9%)
Sum	357 (32,7%)	521 (44,7%)	214 (19,6%)
Difference CL/PL	0.010	0.024	0.900

#### What is your opinion on the indications for aligner treatment?

The opinions of Chileans and Polish differ significantly. Chileans are far more likely to believe in aligner treatment capability than Poles (Table 22).

**Table 22:** Belief in the capabilities of aligners among the respondents

Country/Answer	Any malocclusion can be treated with aligners.	Only non-complex malocclusion can be treated with this method.	I have no idea.
Chile	161 (24,0%)	284 (42,4%)	225 (33,6%)
Poland	28 (6,6%)	224 (33,4%)	170 (40,3%)
Sum	189 (17,3%)	508 (46,5%)	395 (36,2%)
Difference CL/PL	0.001	< 0.001	0.029

**Fig. 19:**
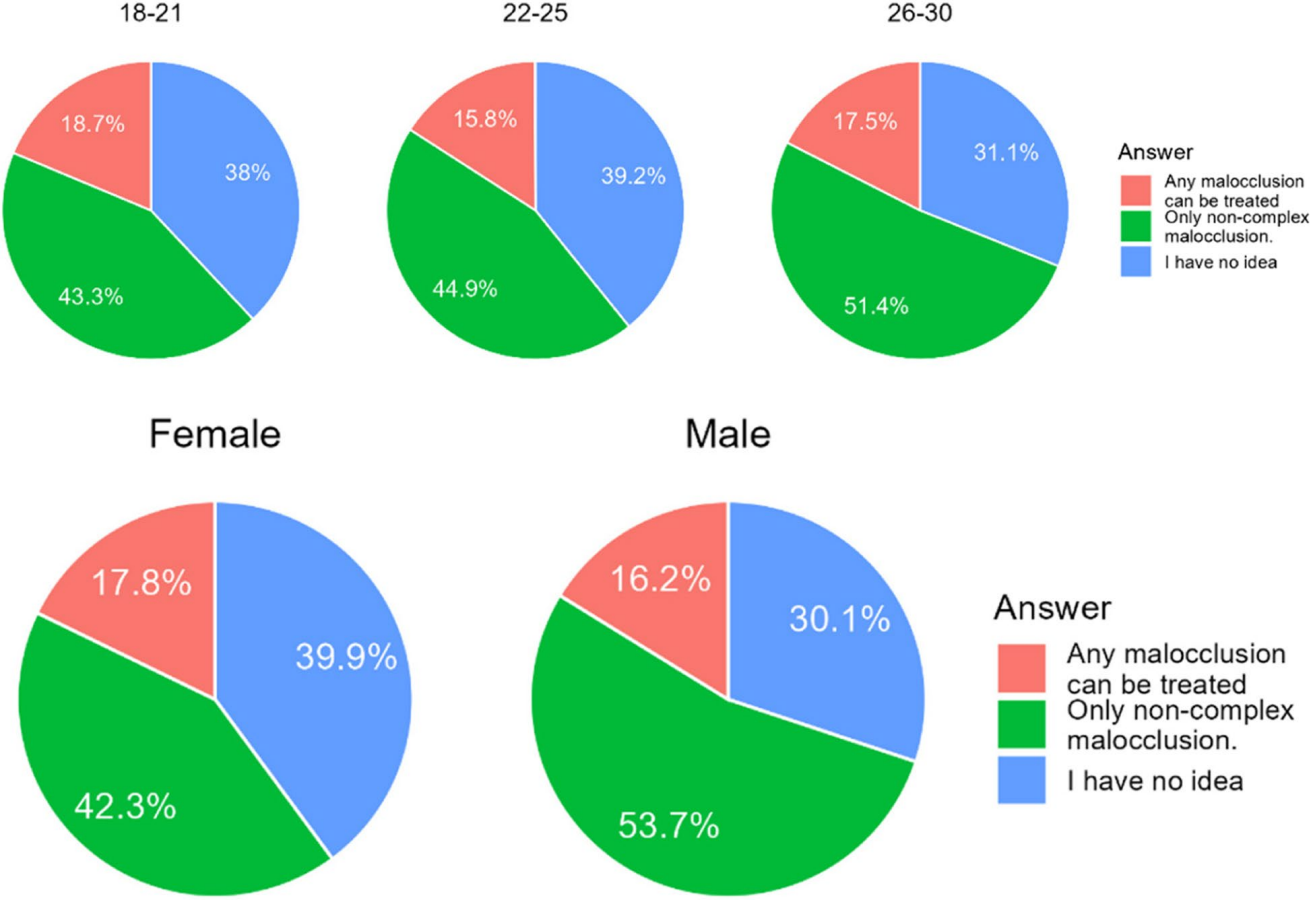
Differences in answers according to age and gender of the respondents

There were no significant differences between the age groups. Female have believed more firmly in the capabilities of aligners than men (Fig. 19).

#### Do you think your doctor can verify that you wear aligners for as long as indicated?

The majority of respondents believe that the doctor is able to see on control visit how long the patient wears the aligners (Table 23). There were no significant correlations regarding age groups or gender.

**Table 23:** Supposition about the doctor’s ability to control the progress of aligner treatment

Country	No	I don’t know	Yes
Chile	26 (3,9%)	170 (25,4%)	474 (70,7%)
Poland	46 (10,9%)	125 (29,6%)	251 (59,5%)
Sum	72 (6,6%)	295 (27,0%)	725 (66,4%)
Difference CL/PL	< 0.001	0.142	< 0.001

#### What do you think about the possible results of aligner treatment?

The majority of the respondents either don’t know the answer on given question or tend to think that it is similar to one with fixed appliance (Table 24). There were no significant correlations regarding age groups or gender.

**Table 24:** Belief in the capabilities of aligners in comparison to standard fixed appliance among the respondents

Country/Answer	Better than fixed braces treatment	I don’t know	Is not as precise as with brackets	They are perfect
Chile	37 (5,5%)	371 (55,4%)	183 (27,3%)	79 (11,8%)
Poland	27 (6,4%)	220 (52,1%)	162 (38,4%)	13 (3,1%)
Sum	64 (5,9%)	591 (54,1%)	345 (31,6%)	92 (8,4%)
Difference CL/PL	0.640	0.325	< 0.001	< 0.001

#### What do you think about the possible treatment time with aligners?

The majority of the respondents don’t know whether aligner treatment is shorter than that with fixed appliance. The second most frequent answer was that the treatment is shorter than fixed treatment. However, above one third claimed that the aligner treatment is either similar or longer than with fixed appliance (Table 25). There were no significant correlations regarding age groups or gender.

**Table 25:** Belief in the capabilities of aligners in comparison to standard fixed appliance among the respondents

Country/Answer	Shorter than fixed braces treatment	I don’t know	Similar to treatment with fixed braces	Longer than fixed braces treatment
Chile	136 (20,3%)	331 (49,4%)	109 (16,3%)	94 (14,0%)
Poland	79 (18,7%)	168 (39,8%)	70 (16,6%)	105 (24,9%)
Sum	215 (19,7%)	499 (45,7%)	179 (16,4%)	199 (18,2%)
Difference CL/PL	0.575	0.002	0.956	< 0.001

## Discussion

People born from 1995 to 2012, who are just entering the labor market, often still during education, are defined as Generation Z (Gen Z) [16]. They are the first people to grow up in a fully computerized society [17]. In fact, young adults were chosen as a target population, as they are beginning to take independent decisions, undertake their first jobs, earn they fist money and create their own images in social media. Moreover, they often find or change partners at this age. The facial appearance and a beautiful smile are very important for this age group [18]. Therefore, as far as young adults are concerned, it can be reliably stated that they are all active in social media and their social life is not possible without social media. Generation Z is the first generation living in a digitized world from the very beginning of their lives. As far as young adults are concerned, it can be reliably stated that they are all active in social media and their social life is not possible without social media. Through a series of associated lifestyle changes, they perceive a range of values differently than people who grew up in a more analog world. This involves several issues related to health and beauty, included orthodontics. Many of Gen Z patients start to seek information on the internet e.g., on social media platforms, before visiting a physician [17]. This information often prompts them to reflect about their health and beauty and take a variety of actions. The authors of the recent study pointed that a pandemic-related increase in the popularity of homeoffice is correlated with significant increase in demand for orthodontic treatment. Nowadays, more people are paying attention to how they look on webcam, so the so-called zoom-boom has directed patients to orthodontic offices to seek for treatment [19]. Moreover, in another novel research, laypeople were asked to assign possible personality traits based on appearance to people with different malocclusions. The malocclusions were classified into five distinct categories by orthodontists according to IOTN. It was proven that traits that are important to succeed in professional life, such as employability, honesty, intelligence, and ability to meet obligations, were assigned significantly more frequently to people with IOTN = 1 [20].

Informatization has not left orthodontics and clinical daily routine. Many procedures are now performed exclusively digitally, the use of specialized software is increasing among physicians, as evidenced by the popularity of software such as Dolphin, Onyxceph, Orthodontics Ortobajt, or Dental Monitoring [14]. One of the symbols of digital revolution is aligner, which since the begging of Invisalign in late nineties, is planned digitally [21]. Digital tools, 3D software, and the evolution of aligners have introduced many innovations to orthodontic care. Orthodontic aligners give the patients a new option of esthetic treatment. Advertisement of the new appliances may attract to orthodontic offices people who desire a more pleasant smile but would never want to have brackets on their teeth. The advantage to eat and brush or floss the teeth without the archwire increases patients’ comfort. Having a modern appliance instead of “old-fashioned” brackets may influence the patients’ social image or position. Patient’s reasons for choosing aligner treatment and knowledge about some aspects of the aligner therapy have been described in a questionnaire study on Arabian patients [22]. This type of appliance is growing in popularity for aesthetic reasons [22]. However, in the present study the invisibility was considered an important characteristic, but more important was the price, treatment time and the expected excellent result. Many clinical trials point out that the use of aligners may be associated to a higher patient satisfaction then standard fixed appliance therapy, as it does not require many lifestyle changes related to choosing the right type and consistency of food, speaking, or discomfort caused by gum irritation [23, 24]. This is consistent with the results of the present study.

The data included in the present study provides information for planning orthodontic care and to better tailor it according to the requirements of future patients.

The perception of orthodontic treatment in society has changed, appearing more accessible. Nowadays, not only severe malocclusions or wealthy patients are treated, as was the case [25]. Moreover, some companies are going out with marketing not only to the doctor, but directly to the prospective patient (as a future customer) in an effort to influence the type of therapy they choose [26]. Both Chile and Poland are developed countries with robustly growing economies, what confirmed HDI 2023 index for Poland is 0,876 and for Chile is 0,855 [27]. A higher percentage of respondents living in big cities in Chile than in Poland reflects the differences in demographic structure of the countries. According to World Bank data, the urbanisation rate in Chile is approximately 89%, compared with only 60% for Poland. The role of the Santiago de Chile agglomeration, in which a great percentage of Chile’s population lives, cannot be overestimated [28].

No standard procedures have been published referring to questioning populations in social media [19]. On the other hand, social media shine as optimal platform to collect a large amount of data in form of surveys. Thus, the authors conducted the study according to the described consensus to maintain the high quality of data collection and presentation [29]. The number of respondents was determined based on the proposed calculations of one of the largest online survey companies, which indicates what is the minimum number of surveys on a given topic in order to consider the results binding for the target group [15]. In the provided link, it is clearly stated that for surveyed groups whose size is more than hundred thousand people, it is necessary to collect 400 respondents in each country.

Interestingly, more Polish than Chilean respondents had never received orthodontic treatment. This indicates that in Poland there may be an increased need for orthodontic treatment in the future, as it has been proven that in adult patients who have never received any orthodontic treatment, IOTN increases with time due to complications of dental misalignment [30, 31]. The demand for orthodontic retreatment seems a very interesting issue in terms of public health. The potential reasons may probably depend on the quality of treatment results or on compromised patient cooperation during orthodontic retention [32].

Poles were more often treated with removable appliances in childhood, while Chileans were most often treated with fixed braces in their teenage years. This may be due to differences in attitudes of physicians towards both removable appliances treatment and functional treatment, which were far less common in America than in Europe [33]. As for knowledge of people who have been treated or are treated with aligners, it is similar in both countries.

Respondents who claimed the willingness to be treated only with aligners constitute the second largest group in this study. This is consistent with the results of the British Orthodontic Society’s 2021 clinical survey, in which it could be noted a significant increase in orthodontic treatment demand, including primarily aligner treatment demand among young adults (18–34) [19, 34].

Differences in familiarity with aligner brands are apparent between countries. The differences in recognition of the Invisaling and DrSmile brands between the countries is probably due to large advertisement campaign by DrSmile. However, lately DrSmile is in Poland referred in numerous press and online articles Most of these articles pertained to customers who were unsatisfied with the service [35, 36]. This could have influenced the opinions of Poles about aligner treatment. In the present study, Polish people are less likely to go on an appointment with mindset to be treated with aligners than Chileans. They do not believe in the capability of treating complex malocclusions with aligners, either, contrary to Chileans.

The finding that in Chile the most important sources of information about the aligners were orthodontists and dentists, whereas in Poland, the most important sources were social media may indicate that Polish practitioners are not strongly promoting aligners. It should be noted that the cost of aligner treatment is also much higher for the doctor. Another reason may be a higher willingness to follow doctors’ recommendations in Chile than in Poland. Interestingly, a study enrolled in Spain found a significant impact of dental service marketing via social media - respondents found that the online image of the practice influenced their decisions on where to seek treatment [37].

In both countries receiving specialty training is a challenging experience, as well as raising the prestige of the physician. In Poland, the post-graduate program lasts 3 years. It is offered mainly at the medical universities, but also in private dental clinics. The program is free of charge; thus, the number of students is strictly limited, only postgraduates with very high results of state dental examination (obligatory for all graduates to receive the license to practice) can participate. However, many general dentists offer orthodontic treatment to patients as it is allowed according to the Polish law. In Chile, the specialization program is offered by both public and private universities and has a duration of 3 years. Enrollment takes place in specific for each institution proposing the postgraduate program. Undertaking specialization training is paid. Upon completion of this program, graduates receive a specialty certification diploma. The entity responsible for certifying this specialization is CONACEO (National Autonomous Corporation for Certification of Dental Specialties). This organization’s primary purpose is to grant certification as a specialist in orthodontics and 11 other dental specialties, all recognized by the Ministry of Health since 2016. The fact that respondents in the middle and upper age subgroups were far more attentive to the title of specialist than younger respondents may reflect a higher understanding of the importance of professional experience and specialized knowledge among young employees comparing to undergraduate students. This may also be due to unawareness, as well as the fact that not all lay people are fully aware of the existence of dental specialities. In a survey carried out among Australians and Swedes, more than 90% of respondents could not clearly distinguish between orthodontist and general dental practitioner [38]. The current survey shows a large group for whom the title of orthodontic specialist is important, but they are in the minority. Adequate education should be provided to the society referring to the importance of the knowledge and experience of specialists in orthodontics. Consultation and treatment by professionals allow to achieve a high standard of health-oriented and esthetic orthodontic treatment.

The finding that, 52.3% would not choose orthodontic treatment in a non-medical setting (based on intraoral scans) at a commercial facility is consistent with results of a recent survey among users of direct-to-consumer (DTC) orthodontics - 50% went to an orthodontist to confirm the need for treatment before proceeding with orthodontic treatment at a DTC; subsequently, more than 80% were satisfied with orthodontic treatment without medical supervision [26]. Similarly, an American study showed that adult patients with a strong motivation for orthodontic therapy tended to prefer an orthodontist, while those with a moderate motivation, a DTC [39]. The impact of DTC orthodontics on the orthodontic market is significant, for example, the American company SmileDirectClub reported in 2018 over 300,000 starts, with an overall value of 3.2 billion dollars [40]. In a British Orthodontic Society survey, 99% respondents want their local medical authority to act against such companies [19, 34]. Generation Z is showing a societal shift in the perception of orthodontic treatment accessibility. This generation is more informed and has greater access to information about orthodontic treatments due to the proliferation of digital technology. They often compare themselves to compare their appearance to other people visible on social media An online survey conducted on laypeople’s perception of orthodontic treatment complexity in USA found that there was a significant inverse association between the complexity of an orthodontic case and the likelihood of choosing DTC treatment over an orthodontist. This suggests that consumers are more likely to choose DTC orthodontics for less complex cases [41]. The rise of DTC orthodontics also brings about implications for patient choice and safety. While DTC orthodontics can be a more affordable and convenient option for some patients, it’s important to note that these services may not be suitable for everyone, especially those with complex orthodontic cases. It is noticeable that in the present study patients in both countries the want to feel safe and thus oppose treatment without supervision by a professional, in contrary to what has been found in the American setting. It is not surprising that most respondents from Chile and Poland put their trust in the doctor’s choice of treatment.

In general, respondents did not have much knowledge regarding aligners. They admitted that they did not know whether aligners could be used in paediatric patients, whether the quality of cooperation on the part of the patient could be checked, or what the effectiveness of aligners was. In this context, the results of the study by Alami et al. [42] and Almotairy et al. [22] are particularly intriguing. The results, similarly to present study, indicated that more than half of aligner-treated patients decided to be treated with aligners already before the first appointment. Thus, the lack of knowledge is not a deterrent to treatment, and mainly aesthetic considerations and a rapid visibility of the first changes are the main factors prompting to choose this type of treatment. On the other hand, 90% respondents in the study by Alami et al. claimed that they considered the information about aligner treatment and the instructions from the doctor to be sufficient [42]. Also in this regard, the clinician’s key role as an intermediary in undertaking a particular type of orthodontic treatment is evident, even in those previously determined to have aligner treatment. This stands in line with the results of the study by Mathew et al. on treatment understanding by patients undergoing treatment: patients undertreatment had more knowledge than the respondents the present study. This indicates the need for medical consultation, patient support and patient education by the physician [43]. On the other hand, it should be underlined that the extent of aligners use varies among orthodontists themselves [44], which undeniably affects the message patients can receive.

The respondents of the present study consider that aligner treatment is a less painful alternative. This claim has been confirmed by a number of studies, including a meta-analysis with a pooled study group of 273 subjects [45]. It should be noted, however, that sharply curved attachments can also be a source of discomfort, including pain [46]. However, the patient wearing an aligner covers the attachments with plastic, which can be a proprioceptive stimulus prompting cooperation and wearing aligners [47].

As the most important characteristics of the future appliance the patients consider: price, treatment time, excellent result, aesthetics of the appliance and comfort. However, many patients admitted that they would pay extra for aligners in the finishing phase, as the final positioning of their teeth would be most important to them. No study could be found comparing such treatment with classical finishing with fixed braces. However, evidence supports aligner as a good alternate to fixed appliances in patients with mild-to-moderate malocclusion [48] Therefore in patients who cannot afford high-quality, expensive, long-lasting aligner treatment finishing with aligners seems an optimal and more economic solution.

The respondents do not have a unified view on how long the doctor should be responsible for maintaining treatment outcomes. On the other hand, clinician orthodontists have a more clearer opinion: more than half of the respondents declared that the retention phase of orthodontic treatment should last a lifetime [49].

The limitation of the present study was that respondents were recruited in social media groups which were somehow associated with the university environment and therefore the study, may overrepresent people with higher education and social status. The need for orthodontic treatment, as determined by standard measures, is influenced by socio-economic status through mechanisms that are not yet fully understood. Another limitation is that the questionnaire form was validated on the small group of the respondents and the low response rate, which may indicate that people with more interest in the topic participated in the survey.

## Conclusions

Despite the geographical and cultural differences between countries, tendencies emerging in patients’ perception of orthodontic treatment can be clearly identified.

1. In both countries, patients rely on orthodontists for the choice of appliance, however there is a high percentage of patients who want to be treated exclusively with aligners. They usually demand to be treated and monitored by an orthodontist.
2. Aligners are more favoured In Chile than in Poland. Direct-to-consumer orthodontics does not seem attractive to patients.
3. Despite advertisements in social media young adults do not have adequate knowledge about aligner treatment. It should be underlined that patients are characterised by a limited understanding of orthodontic treatment and should be under care of a specialist.
4. Many people want to be treated despite a previous orthodontic treatment. Almost one in three people would like to seek orthodontic treatment in the nearest future.

### Supplementary Information

**Additional file 1:.** S1 – Scan of decision of the bioethical committee of Pomeranian Medical University in Szczecin

## Data Availability

The data is available from corresponding author upon reasonable request.

## Abbreviations

IOTN: Index of orthodontic treatment need
ICON: Index of complexity outcome, and need
DAI: Dental aesthetic index
TPI: Treatment priority index
DHC: Dental health component
AC: Esthetic component
CL: Chile
PL: Poland
DTC: Direct-to-consumer
