# Supplementary material for: Orthodontic treatment demand for fixed treatment and aligners among young adults in middle Europe and South America – a questionnaire study
Source: BMC Oral Health. 2024 Mar 2;24:292. doi: 10.1186/s12903-024-04023-0 (PMC10908024; doi:10.1186/s12903-024-04023-0)
Supplement: Supplementary file 1 — Additional file 1:. S1 – Scan of decision of the bioethical committee of Pomeranian Medical University in Szczecin [file 12903_2024_4023_MOESM1_ESM.pdf]

Pomorski Uniwersytet Medyczny w Szczecinie

KOMISJA BIOETYCZNA

Szczecin, 05 września 2022 r.

KB.006.83.2022/Z-6546

Pan
Maciej Jedliński
Zakład Stomatologii Zintegrowanej PUM

Szanowny Panie

W związku ze zgłoszeniem do Komisji Bioetycznej Pomorskiego Uniwersytetu Medycznego w Szczecinie opisu badania pt *„Co młodzi dorośli myślą o leczeniu nakładkami? - badanie ankietowe w krajach Europy i Ameryki Łacińskiej*", sygn. RPW/6546/2022P uprzejmie informuję, iż z przedłożonej dokumentacji wynika, że przedmiotowe badanie nie wymaga opinii Komisji Bioetycznej.

Z poważaniem

PRZEWODNICZĄCY
Komisji Bioetycznej
Pomorskiego Uniwersytetu Medycznego
w Szczecinie

prof. dr hab. n. med. Marek Droździk

Certified translation from Polish

[logo]

Pomeranian Medical University in Szczecin
(Pomorski Uniwersytet Medyczny w Szczecinie)

BIOETHICS COMMITTEE

KB.006.83.2022/Z-6546

Szczecin, 5th September, 2022

Mr Maciej Jedliński
Department of Integrated Dentistry
Pomeranian Medical University in Szczecin

Dear Sir,

Following the notification of description of the study entitled: *"What do young adults think about aligner treatment? - A survey in European and Latin American countries"*, ref. RPW/6546/2022P, to the Bioethics Committee of Pomeranian Medical University in Szczecin, please kindly be informed that the documentation provided to the Committee shows that the above study does not require opinion of the Bioethics Committee.

Regards,

/-/Impression of a seal, reading: "President of the Bioethics Committee of Pomeranian Medical University in Szczecin, Prof. dr hab. n. med. Marek Droździk"
/-/Personal signature

Certified to be a true and exact translation of the original document from Polish.

Repertory number: 1424/2023 Szczecin, 14.11.2023

JACEK GOSK TŁUMACZ PRZYSIĘGŁY JĘZYKA ANGIELSKIEGO NR TP/500/05

J. Gosk

SWORN TRANSLATOR
AND
INTERPRETER OF ENGLISH
Jacek Gosk, M.A.
70-402 Szczecin, ul. Kaszubska 57
phone: +(4891) 4336250, mobile: 0605 935934
